# PLPP/CIN-mediated NF2-serine 10 dephosphorylation regulates F-actin stability and Mdm2 degradation in an activity-dependent manner

**DOI:** 10.1038/s41419-020-03325-9

**Published:** 2021-01-04

**Authors:** Ji-Eun Kim, Duk-Shin Lee, Tae-Hyun Kim, Hana Park, Min-Ju Kim, Tae-Cheon Kang

**Affiliations:** Department of Anatomy and Neurobiology, Institute of Epilepsy Research, College of Medicine, Hallym University, Chuncheon, 24252 South Korea

**Keywords:** Cell death in the nervous system, Epilepsy

## Abstract

Neurofibromin 2 (NF2, also known as merlin) is a tumor suppressor protein encoded by the neurofibromatosis type 2 gene *NF2*. NF2 is also an actin-binding protein that functions in an intrinsic signaling network critical for actin dynamics. Although protein kinase A (PKA)-mediated NF2-serin (S) 10 phosphorylation stabilizes filamentous actin (F-actin), the underlying mechanisms of NF2-S10 dephosphorylation and the role of NF2 in seizures have been elusive. Here, we demonstrate that pyridoxal-5′-phosphate phosphatase/chronophin (PLPP/CIN) dephosphorylated NF2-S10 site as well as cofilin-S3 site. In addition, NF2-S10 dephosphorylation reversely regulated murine double minute-2 (Mdm2) and postsynaptic density 95 (PSD95) degradations in an activity-dependent manner, which increased seizure intensity and its progression in response to kainic acid (KA). In addition, NF2 knockdown facilitated seizure intensity and its progress through F-actin instability independent of cofilin-mediated actin dynamics. Therefore, we suggest that PLPP/CIN may be a potential therapeutic target for epileptogenesis and NF2-associated diseases.

## Introduction

Neurofibromin 2 [NF2, also known as merlin (moesin-ezrin-radixin-like protein) or schwannomin] is a tumor suppressor protein encoded by the neurofibromatosis type 2 gene *NF2*. Deletion or loss-of-function mutation of *NF2* causes neurofibromatosis type 2, which is a dominant inherited disorder characterized by the development of multiple benign tumors in the nervous system. The most common tumors found in neurofibromatosis type 2 are schwannoma, meningioma, and ependymoma^1–3^. NF2 is also an actin-binding protein that links membrane proteins to cytoskeleton and functions in an intrinsic signaling network critical for actin dynamics in various cells^4–6^.

Filamentous actin (F-actin) plays an important role in stabilization and structural modification of dendritic spines that are critical structural and functional components of neurons receiving and integrating the majority of excitatory synaptic inputs^7–11^. Indeed, F-actin polymerization (by jasplakinolide) increases seizure threshold response to picrotoxin, while depolymerization (by latrunculin A) decreases it^12^. In the brain, NF2 expresses in dendrites^13^, axons^14,15^, the cytoplasm^16,17^, and synaptic junctions^18^ of cortical and hippocampal neurons. Interestingly, c.428_430delCTTdel mutation in *Nf2* is associated with a predisposition to development of benign brain tumors in which the on-set of symptoms is characterized by status epilepticus (SE, a prolonged seizure activity) in humans^19^. Furthermore, valproic acid (an anti-epileptic drug) up-regulates NF2 expression^20^. With respect to NF2–actin interactions^4–6^. it is likely that NF2-mediated actin dynamics would play an important role in the regulation of neuronal excitability. However, the role of NF2 in F-actin stabilization is still controversial, although NF2 is involved in actin dynamics. NF2 stabilizes F-actin and reduces its depolymerization rates^21^. In contrast, loss of function of NF2 inhibits F-actin severing and depolymerizing activity of cofilin by increasing LIM domain kinase-1 (LIMK1)-mediated serine (S) 3 phosphorylation^22,23^.

On the other hand, NF2 activity is regulated by phosphorylations. C-terminal S518 site is phosphorylated both by protein kinase A (PKA) and p21-activated kinase (PAK)^24,25^. Phosphorylation at this residue inhibits NF2 tumor suppressor activity by blocking its head-to-tail interaction^24^, which leads to cell growth and cell division^26,27^. Indeed, S518 dephosphorylation by the myosin phosphatase activates NF2 that promotes growth arrest and neurite outgrowth^13,26,28^. S518 phosphorylation also weakens the NF2-cytoskeleton associations^24^. In contrast, PKA-mediated NF2-S10 phosphorylation stabilizes actin filaments^29^. Therefore, the selective phosphorylations at S10- and S518 site play reverse roles in NF2-associated actin dynamics. However, little is known yet to explain the underlying mechanisms of NF2-S10 dephosphorylation and the role of NF2 phosphorylation in seizure susceptibility and/or epilepsy.

Pyridoxal-5′-phosphate phosphatase/chronophin (PLPP/CIN) is firstly discovered as a phosphatase for pyridoxal-5′-phosphate (PLP, an active form of vitamin B_6_)^30^, and later identified as a serine protein phosphatase of the non-thiol-based haloacid dehalogenase superfamily of hydrolases and a modulator for cofilin activity^10,11,31–33^. Furthermore, we have recently reported that PLPP/CIN directly dephosphorylates calsenilin (CSEN, also referred as downstream regulatory element antagonist modulator and potassium channel interacting protein 3) and neuronal precursor cell expressed developmentally downregulated 4-2 (NEDD4-2), which are involved in the regulations of *N*-methyl-*D*-aspartate receptor (NMDAR) and α-amino-3-hydroxy-5-methyl-4-isoxazolepropionic acid receptor (AMPAR) functions, respectively^34,35^. PLPP/CIN also dephosphorylates murine double minute-2 (Mdm2, an E3-ubiquitin ligase) at S166 site, which inhibits the degradation of postsynaptic density 95 (PSD95) during activity-dependent synapse eliminations^36–38^. Interestingly, the N-terminal region of NF2 is responsible for the direct NF2-mediated Mdm2 degradation, which is not related to the Mdm2 transcriptional repression^39^. Considering the roles of NF2 in actin dynamics and Mdm2 regulation^4–6,21,39^, it is noteworthy to elucidate whether interaction of PLPP/CIN with NF2 modulates actin dynamics and Mdm2 degradation in neuronal excitability.

Here, we demonstrate that PLPP/CIN bound to NF2 and dephosphorylated its S10 site without altering PKA activity, which reduced F-actin stability. In addition, NF2-S10 dephosphorylation reversely regulated Mdm2 and PSD95 degradations in an activity-dependent manner, which increased seizure intensity and its progression in response to kainic acid (KA). NF2 knockdown facilitated KA-induced seizure activity through F-actin instability and the reductions in Mdm2 and PSD95 degradation, independent of cofilin activity. These findings indicate that PLPP/CIN may increase F-actin instability and NF2-mediated Mdm2 degradation, but inhibit PSD95 elimination, by dephosphorylating NF2-S10 and Mdm2-S166 site, which lead to neuronal hyperexcitability. Therefore, we suggest that PLPP/CIN may be a potential therapeutic target for epileptogenesis and NF2-associated diseases.

## Results

### PLPP/CIN directly binds to NF2 and dephosphorylates S10 site in vitro and in vivo

**Fig. 1:**
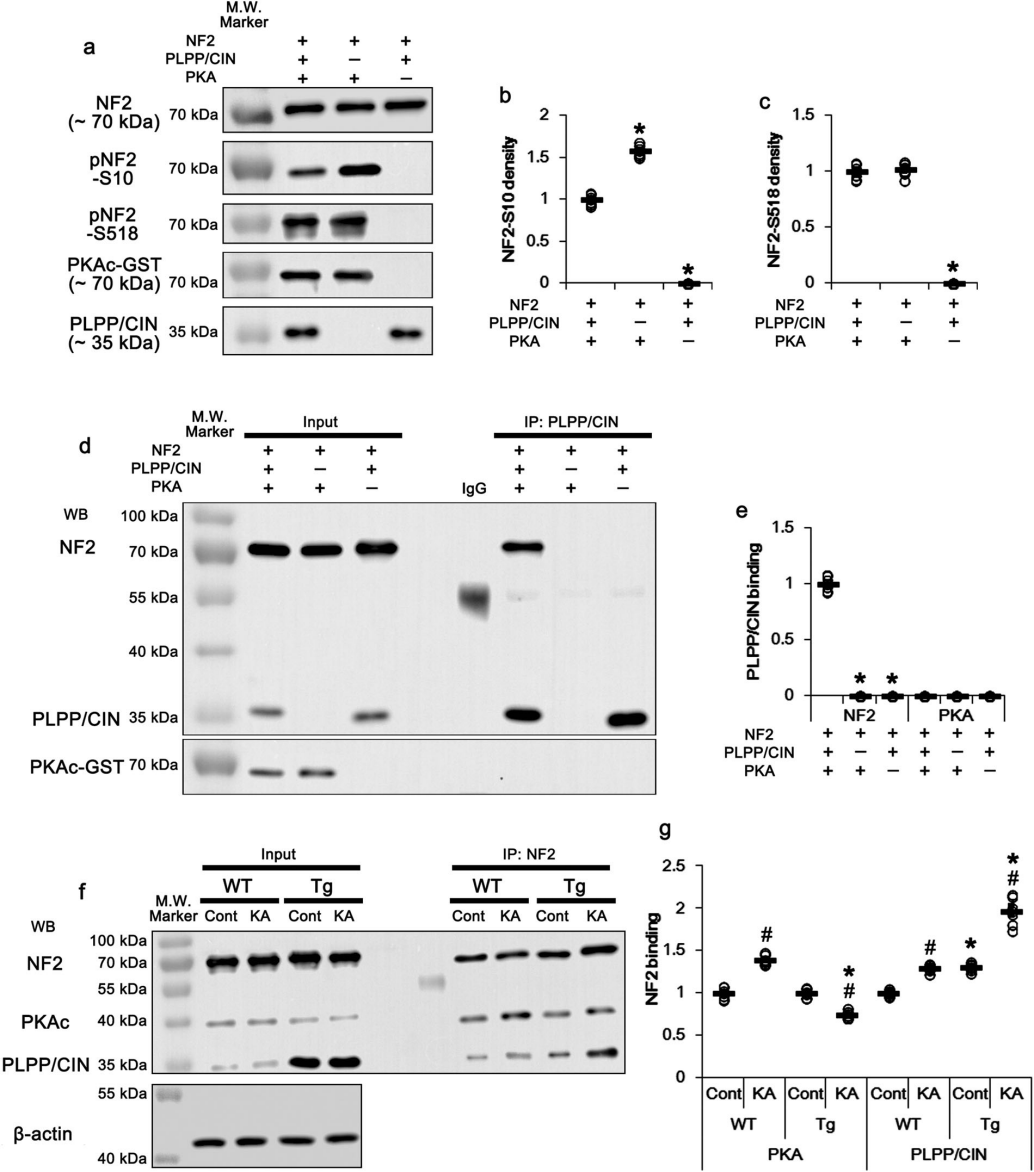
PLPP/CIN-mediated NF2 dephosphorylation. **a**–**e** In vitro assay using recombinant proteins. **a** Representative western blot data demonstrating PLPP/CIN-mediated NF2 dephosphorylation. **b, c** Quantification of NF2-S10 (**b**) and -518 (**c**) phosphorylation levels based on western blot data. Open circles indicate each individual value. Horizontal bars indicate mean value (*p < 0.05 vs. the presence of PLPP/CIN and PKAc; *n* = 7). **d** Representative co-immunoprecipitation data demonstrating PLPP/CIN-NF2 and -PKAc bindings. **e** Quantification of PLPP/CIN-NF2 and -PKAc bindings. Open circles indicate each individual value. Horizontal bars indicate mean value (*p < 0.05 vs. the presence of PLPP/CIN and PKAc; *n* = 7). **f**, **g** Effects of KA on PLPP/CIN-NF2 and -PKAc bindings in WT and *PLPP/CIN*^*Tg*^ mice in vivo. **f** Representative co-immunoprecipitation data demonstrating PLPP/CIN-PKAc and NF2 bindings. **g** Quantification of PLPP/CIN-PKAc and -NF2 bindings. Open circles indicate each individual value. Horizontal bars indicate mean value (*^,#^*p* < 0.05 vs. WT and control animals, respectively; *n* = 7).

First, we performed in vitro assay using recombinant proteins to clarify the direct phosphatase activity of PLPP/CIN on NF2. Consistent with previous studies demonstrating PKA-mediated NF2-S10 and -S518 phosphorylations^24,25,29^, NF2-S10 phosphorylation was increased by PKA catabolic subunit (PKAc) (*F*_(2,18)_ = 1541.7, *p* < 0.00001, one-way ANOVA, *n* = 7, respectively; Fig. 1a, b). PLPP/CIN decreased NF2-S10 phosphorylation to ~0.63-fold of vehicle level (*F*_(2,18)_ = 1541.7, *p* < 0.00001, one-way ANOVA, *n* = 7, respectively; Fig. 1a, b). PKAc also increased NF2-S518 phosphorylation (*F*_(2,18)_ = 972.5, *p* < 0.00001, one-way ANOVA, *n* = 7, respectively; Fig. 1a and c). However, PLPP/CIN did not affect PKAc-mediated NF2-S518 phosphorylation (Fig. 1a, c). Furthermore, co-immunoprecipitation demonstrated that PLPP/CIN bound to NF2, when PKAc was present (*F*_(2,18)_ = 1721.3, *p* < 0.00001, one-way ANOVA, *n* = 7, respectively; Fig. 1d and e). However, PLPP/CIN did not bind with PKAc (Fig. 1d, e). These findings indicate that PLPP/CIN may directly bind to NF2 and dephosphorylate its S10 site. In vivo study demonstrated that no differences in NF2-PKAc binding between WT and *PLPP/CIN*^*Tg*^ mice under physiological conditions (Fig. 1f, g). However, *PLPP/CIN*^*Tg*^ mice showed the increased NF2-PLPP/CIN binding to ~1.3-fold WT mice level (*t*_(12)_ = 14.04, *p* < 0.00001, Student’s *t*-test, *n* = 7, respectively; Fig. 1f, g). Together with in vitro assay, these findings suggest that PLPP/CIN may dephosphorylate NF2 at S10 site.

### PLPP/CIN over-expression facilitates NF2-S10 dephosphorylation under physiological- and post-ictal conditions in vivo

**Fig. 2:**
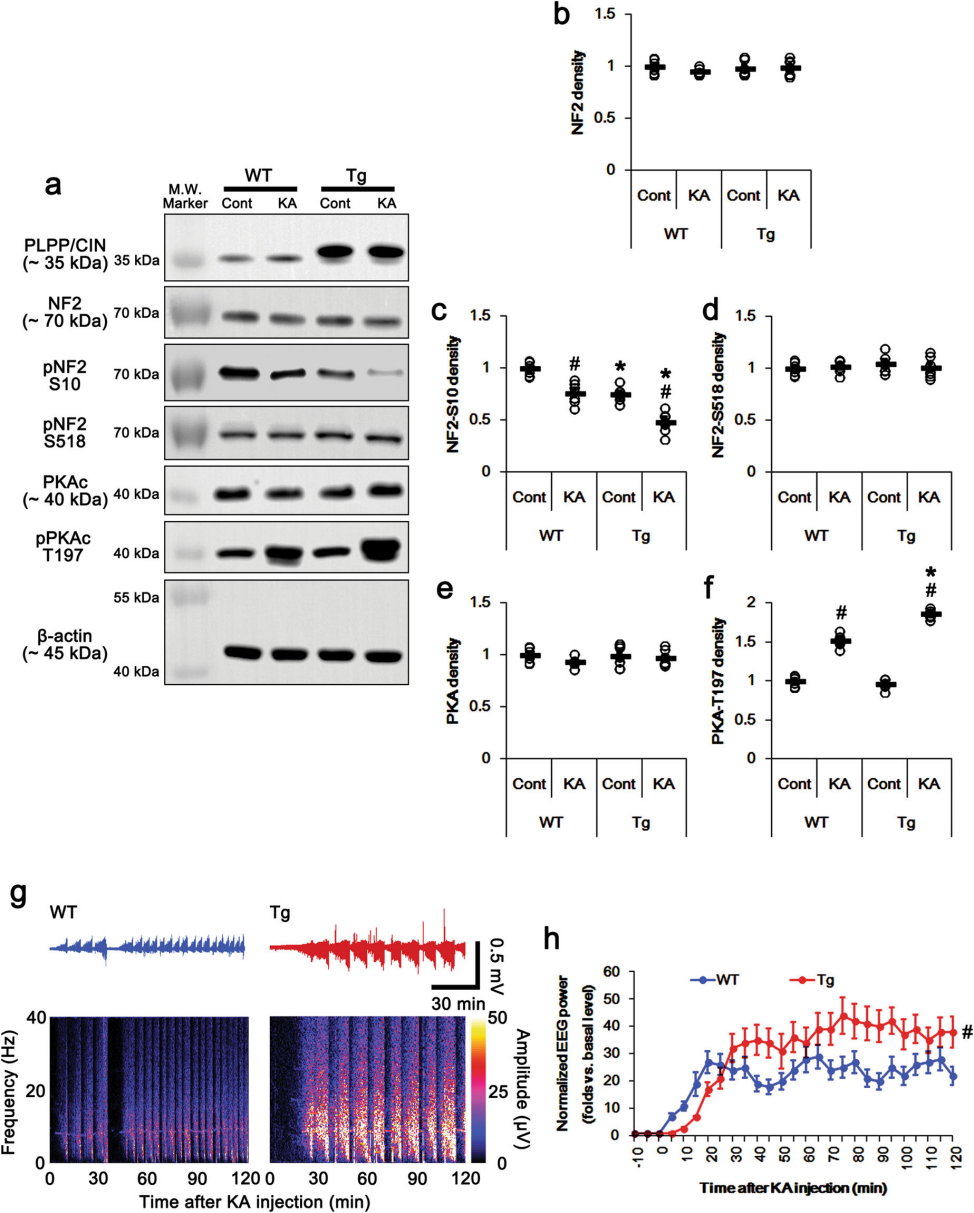
Effects of PLPP/CIN over-expression on NF2 phosphorylation and seizure activity in response to KA. **a** Representative western blots of PLPP/CIN, NF2, pNF2-S10, pNF2-S518, PKAc, and pPKAc in WT and and *PLPP/CIN*^*Tg*^ mice in vivo. **b–f** Quantification of NF2, pNF2-S10, pNF2-S518, PKAc, and pPKAc levels. Open circles indicate each individual value. Horizontal bars indicate mean value (*^,#^*p* < 0.05 vs. WT and control animals, respectively; *n* = 7). **g** Representative EEG traces and frequency–power spectral temporal maps in response to KA. **h** Quantification of total EEG power (seizure intensity) in response to KA (^#^*p* < 0.05 vs^.^ WT animals; *n* = 7, respectively).

Next, we explored whether PLPP/CIN regulates NF2-S10 phosphorylation level in the *PLPP/CIN*^*Tg*^ mouse hippocampus under physiological condition. PLPP/CIN over-expression did not affect total NF2 protein and its S518 phosphorylation levels. However, PLPP/CIN over-expression reduced NF2-S10 phosphorylation level to ~75% of WT mice level (*t*_(12)_ = 6.97, *p* = 0.000015, Student’s *t*-test, *n* = 7, respectively; Fig. 2a–d). PKAc activity (phosphorylation) was unaffected by PLPP/CIN over-expression (Fig. 2a, e, f).

To investigate the effect of seizure activity on NF2 phosphorylations, we injected kainic acid (KA, 25 mg/kg, i.p.). Consistent with previous studies^34,35^, PLPP/CIN over-expression showed the increases in the latency of seizure on-set (*t*_(12)_ = 3.987, *p* = 0.002, Student’s *t*-test, *n* = 7, respectively; Fig. 2g, h) and seizure intensity (severity) in response to KA (*F*_(1,12)_ = 9.698, *p* = 0.009, repeated measures one-way ANOVA, *n* = 7, respectively; Fig. 2g, h). KA decreased NF2-S10 phosphorylation to ~0.76-fold of basal level in WT mice without altering its expression and S518 phosphorylation (*F*_(3,24)_ = 44.11, *p* = 0.0002, one-way ANOVA, *n* = 7, respectively; Fig. 2a–d). However, KA elevated phospho (p)-PKAc level to ~1.5-fold of basal level in WT mice (*F*_(3,24)_ = 324.2, *p* < 0.00001, one-way ANOVA, *n* = 7, respectively; Fig. 2a, e and f). In *PLPP/CIN*^*Tg*^ mice, KA decreased NF2-S10 phosphorylation to ~0.48-fold of basal level without changing total NF2 protein and its S518 phosphorylation levels (*F*_(3,24)_ = 44.11, *p* < 0.00001, one-way ANOVA, *n* = 7, respectively; Fig. 2a–d). In contrast, KA increased pPKAc level in *PLPP/CIN*^*Tg*^ mice more than WT mice (~1.86-fold of basal level; *F*_(3,24)_ = 324.2, *p* < 0.00001, one-way ANOVA, *n* = 7, respectively; Fig. 2a, e and f). KA increased NF2-PKAc bindings to ~1.39-fold of control animal levels in WT mice (*F*_(3,24)_ = 182.3, *p* < 0.00001, one-way ANOVA, *n* = 7, respectively; Fig. 1f, g). In addition, KA enhanced NF2-PLPP/CIN binding to ~1.31-fold of control animal levels in WT mice (*F*_(3,24)_ = 157.1, *p* < 0.00001, one-way ANOVA, *n* = 7, respectively; Fig. 1f, g). In *PLPP/CIN*^*Tg*^ mice, KA reduced the NF2-PKAc binding to ~75% of control animal level (*F*_(3,24)_ = 182.3, *p* < 0.00001, one-way ANOVA, *n* = 7, respectively; Fig. 1f, g), but increased NF2-PLPP/CIN binding to ~1.97-fold of control animal level (*F*_(3,24)_ = 157.1, *p* < 0.00001, one-way ANOVA, *n* = 7, respectively; Fig. 1f, g). These findings indicate that PLPP/CIN-mediated NF2-S10 dephosphorylation may be inversely correlated to seizure activity in response to KA.

### PLPP/CIN deletion attenuates seizure activity and NF2-S10 dephosphorylation in response to KA

**Fig. 3:**
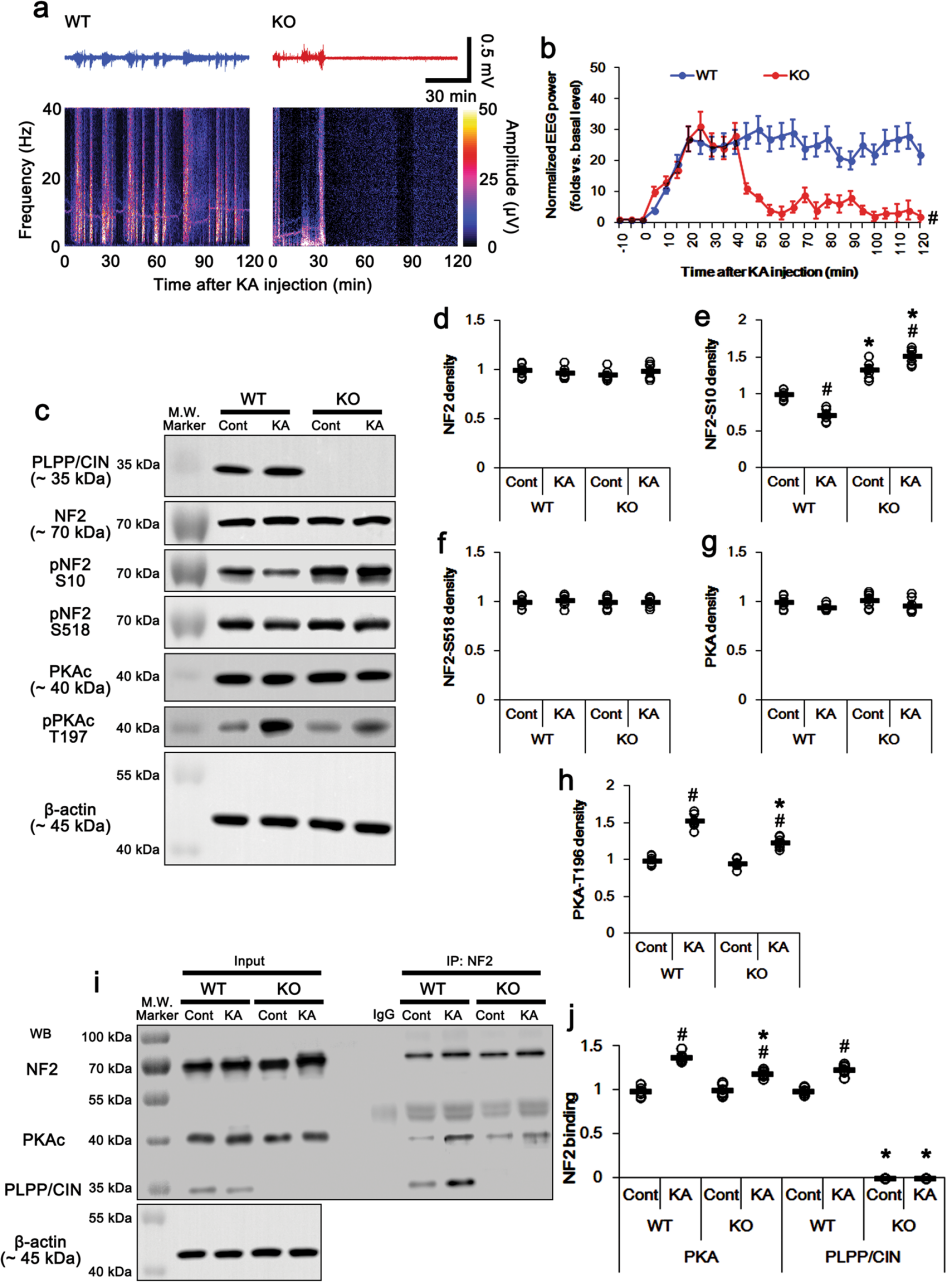
Effects of PLPP/CIN deletion on seizure activity, NF2 phosphorylation, and PLPP/CIN-NF2 binding in response to KA. **a** Representative EEG traces and frequency-power spectral temporal maps in response to KA. **b** Quantification of total EEG power (seizure intensity) in response to KA (^#^*p* < 0.05 vs. WT animals; *n* = 7, respectively). **c** Representative western blots of PLPP/CIN, NF2, pNF2-S10, pNF2-S518, PKAc, and pPKAc in WT and and *PLPP/CIN*^*Tg*^ mice in vivo. **d**–**h** Quantification of NF2, pNF2-S10, pNF2-S518, PKAc, and pPKAc levels. Open circles indicate each individual value. Horizontal bars indicate mean value (*^,#^*p* < 0.05 vs. WT and control animals, respectively; *n* = 7). **i**, **j** Effects of KA on PLPP/CIN-NF2 and -PKAc bindings in WT and *PLPP/CIN*^*−/−*^ mice in vivo. **i** Representative co-immunoprecipitation data demonstrating PLPP/CIN-PKAc and NF2 bindings. **j** Quantification of PLPP/CIN-PKAc and -NF2 bindings. Open circles indicate each individual value. Horizontal bars indicate mean value (*^,#^*p* < 0.05 vs. WT and control animals, respectively; *n* = 7).

To confirm further the role of PLPP/CIN in NF2-S10 dephosphorylation, we investigated the effect of PLPP/CIN deletion on NF2 phosphorylation level in vivo. Consistent with previous studies^34,35^, PLPP/CIN deletion showed the reductions in the latency of seizure on-set (*t*_(12)_ = 6.32, *p* < 0.0001, Student’s *t*-test, *n* = 7, respectively; Fig. 3a, b), and seizure intensity/duration in response to KA (*F*_(1,12)_ = 21.37, *p* = 0.0006, repeated measure one-way ANOVA, *n* = 7, respectively; Fig. 3a, b). Under physiological condition, PLPP/CIN deletion did not affect total NF2 protein, NF2-S518 phosphorylation, and PKAc activity (Fig. 3c–h). However, PLPP/CIN deletion increased NF2-S10 phosphorylation to ~1.52-fold of WT mice level under physiological condition (*t*_(12)_ = 6.86, *p* = 0.00002, Student’s *t*-test, *n* = 7, respectively; Fig. 3c–h). PLPP/CIN deletion enhanced NF2-S10 phosphorylation induced by KA without altering its expression and S518 phosphorylation (*F*_(3,24)_ = 105.4, *p* = 0.007, one-way ANOVA, *n* = 7, respectively; Fig. 3c–f). KA elevated pPKAc level in *PLPP/CIN*^*−/−*^ mice less than WT mice (*F*_(3,24)_ = 90.83, *p* = 0.00003, one-way ANOVA, *n* = 7, respectively; Fig. 3c, g and h). Co-immunoprecipitation revealed that no differences in NF2-PKAc binding between WT and *PLPP/CIN*^*−/−*^ mice under physiological conditions (Fig. 3i, j). Following KA injection, the NF2-PKAc binding was increased to ~1.4-fold of control animal level in WT mice (*F*_(3,24)_ = 78.25, *p* < 0.0001, one-way ANOVA, *n* = 7, respectively; Fig. 3i, j). KA also increased the NF2-PKAc binding to ~1.2-fold of control animal level in *PLPP/CIN*^*−/−*^ mice, which was ~86% of WT mice level (*F*_(3,24)_ = 78.25, *p* < 0.0001, one-way ANOVA, *n* = 7, respectively; Fig. 3i, j). Considering the data obtained from *PLPP/CIN*^*Tg*^ mice, these findings indicate that PLPP/CIN deletion may abolish NF2-S10 dephosphorylation in response to KA, in spite of the lower NF2-PKAc binding and PKAc activity than WT mice, and that PKAc phosphorylation may correlate to seizure intensity. Therefore, our findings suggest that PLPP/CIN may be a counterpart of PKAc for NF2-S10 phosphorylation.

### NF2-S10 phosphorylation correlates with F-actin stability independent of cofilin activity

**Fig. 4:**
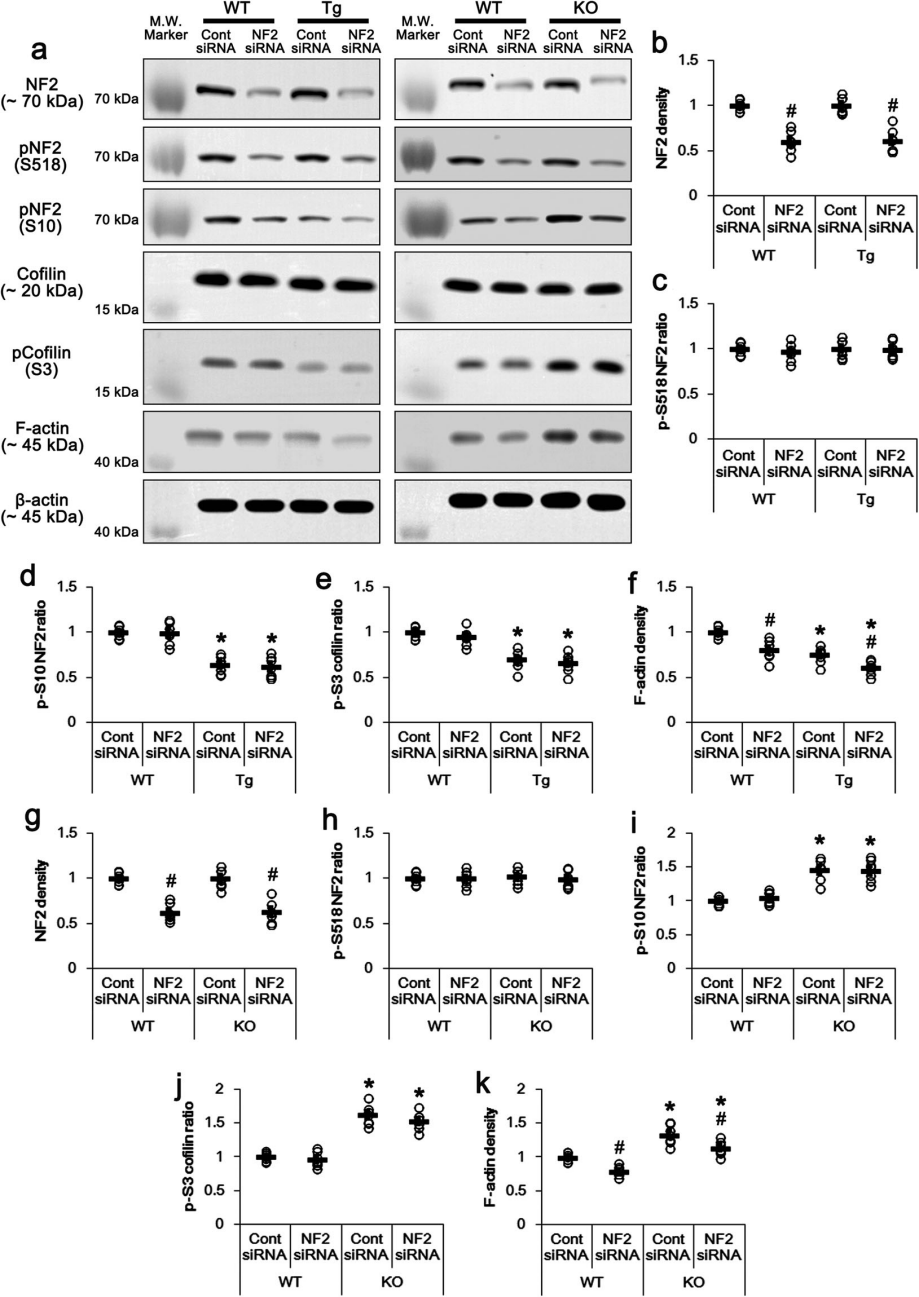
Effects of NF2 knockdown on NF2 and cofilin phosphorylations in *PLPP/CIN*^*Tg*^ and *PLPP/CIN*^*−/−*^ mice under physiological condition. **a** Representative western blots of NF2, pNF2-S10, pNF2-S518, cofilin, pCofilin-S3, and F-actin. **b–k** Quantification of NF2, pNF2-S10, pNF2-S518, cofilin, pCofilin-S3, and F-actin levels based on western blot data. Open circles indicate each individual value. Horizontal bars indicate mean value (*^,#^*p* < 0.05 vs. WT and control siRNA-treated animals, respectively; *n* = 7).

PLPP/CIN facilitates F-actin severing by cofilin dephosphorylation (activation)^31^. Interestingly, NF2 slows filament disassembly with no influence on the assembly rate by binding periodically along F-actin filaments^21^. Furthermore, NF-S10 phosphorylation increases NF2-actin binding and F-actin stability^29^, although S518 phosphorylation weakens the NF2-cytoskeleton associations^24^. However, NF2 deletion facilitates F-actin polymerization by increasing Ras-related C3 botulinum toxin substrate 1 (Rac1)-mediated cofilin phosphorylation^23^. Therefore, we investigated the effects of NF2 knockdown on cofilin-mediated F-actin polymerization. NF2 knockdown effectively reduced total NF2 protein level to 0.61-fold of control siRNA level in WT mice, but not its S10- and S518 phosphorylation ratios (p-protein/total protein), cofilin expression, and cofilin-S3 phosphorylation ratio (*t*_(12)_ = 12.23, *p* < 0.0001, Student’s *t*-test, *n* = 7, respectively; Fig. 4a–e and g–j). In addition, NF2 siRNA reduced F-actin contents to 0.8-fold of control siRNA level in WT mice (*t*_(12)_ = 6.84, *p* = 0.002, Student’s *t*-test, *n* = 7, respectively; Fig. 4a, f and k).

Consistent with previous studies^11,34^, cofilin phosphorylations were 0.7-fold of WT animals in *PLPP/CIN*^*Tg*^ mice (*t*_(12)_ = 6.75, *p* < 0.0001, Student’s *t*-test, *n* = 7, respectively; Fig. 4a and e). In addition, F-actin contents were 0.75-fold of WT animals in *PLPP/CIN*^*Tg*^ mice (*t*_(12)_ = 6.04, *p* < 0.0001, Student’s *t*-test, *n* = 7, respectively; Fig. 4a and f). NF2 knockdown did not affect cofilin phosphorylation level in *PLPP/CIN*^*Tg*^ mice (Fig. 4a, e). However, NF2 siRNA reduced F-actin contents were reduced to 0.61-fold of control siRNA level in *PLPP/CIN*^*Tg*^ mice (*F*_(3,24)_ = 24.6, *p* = 0.008, one-way ANOVA, *n* = 7, respectively; Fig. 4a and f). In *PLPP/CIN*^*−/−*^ mice, cofilin phosphorylations were 1.62-fold of WT animals (*t*_(12)_ = 10.17, *p* < 0.0001, Student’s *t*-test, *n* = 7, respectively; Fig. 4a and j). F-actin contents were 1.33-fold of WT animals in *PLPP/CIN*^*−/−*^ mice (*t*_(12)_ = 5.32, *p* = 0.0001, Student’s *t*-test, *n* = 7, respectively; Fig. 4a and j). Although NF2 knockdown did not affect cofilin phosphorylation level, it reduced F-actin contents were reduced to 1.13-fold of control siRNA-treated WT mice level in *PLPP/CIN*^*−/−*^ mice (*F*_(3,24)_ = 34.6, *p* < 0.00001, one-way ANOVA, *n* = 7, respectively; Fig. 4a and k).

**Fig. 5:**
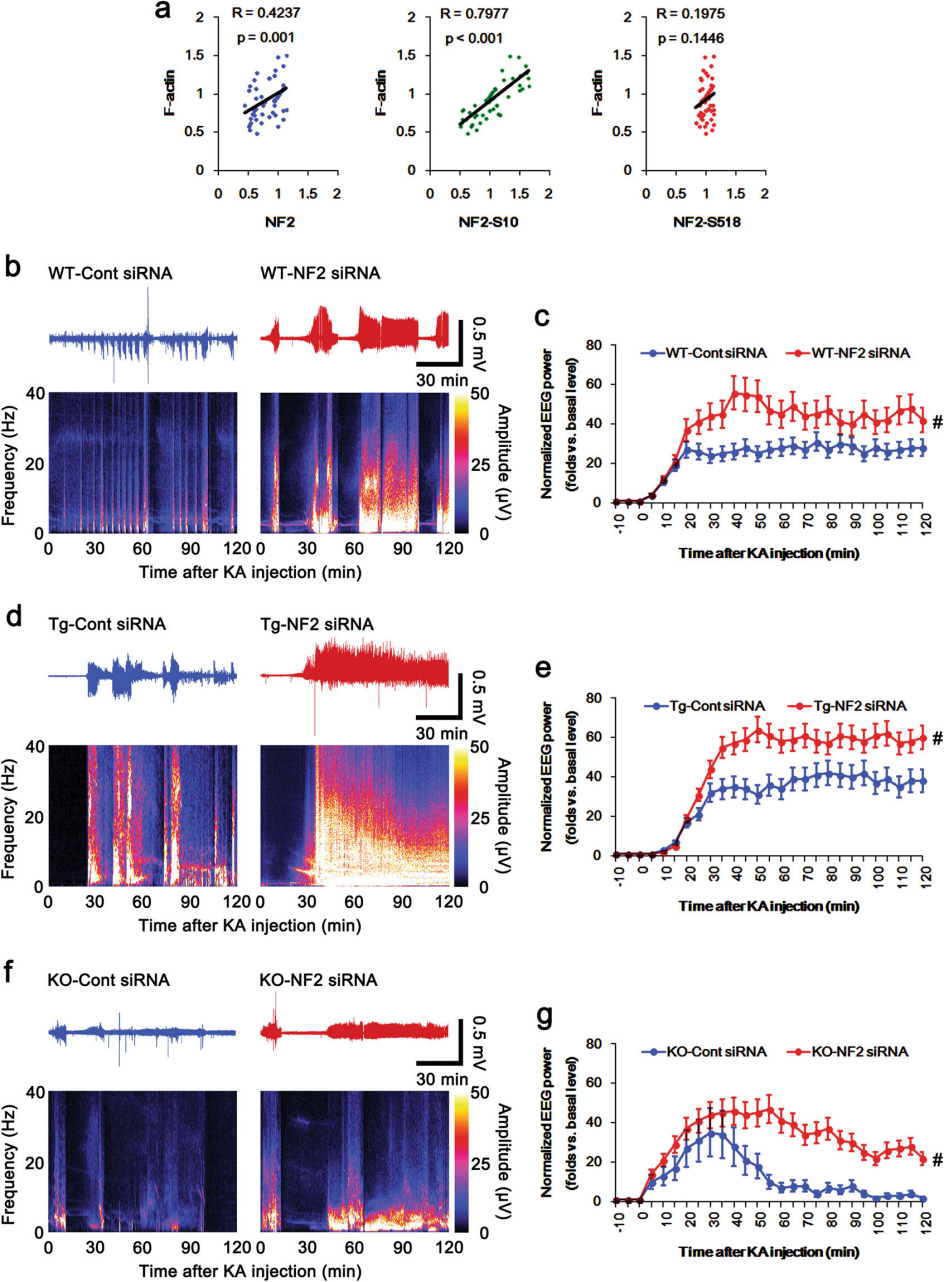
Linear regression analysis between NF2 expression and F-actin contents and the effects of NF2 knockdown on seizure activity in response to KA in *PLPP/CIN*^*Tg*^ and *PLPP/CIN*^*−/−*^ mice. **a** Linear regression analysis between NF2 expression and F-actin contents within control siRNA- and NF2 siRNA-treated groups of WT, *PLPP/CIN*^*Tg*^ and *PLPP/CIN*^*−/−*^ mice under physiological conditions. **b–g** Representative EEG traces and frequency-power spectral temporal maps (**b**, **d**, **f**) and quantification of total EEG power (seizure intensity) in response to KA (**c**, **e**, **g**) in WT (**b**, **c**), *PLPP/CIN*^*Tg*^ (**d**, **e**) and *PLPP/CIN*^*−/−*^ (**f**, **g**) mice (^#^*p* < 0.05 vs. control siRNA-treated animals; *n* = 7, respectively).

Since PKA simultaneously phosphorylates NF2 at S10 and S518 sites^29,40^, we could not directly investigate the effect of the modulation of PKA activity on NF2-S10 phosphorylation. However, PLPP/CIN over-expression and its deletion selectively affected NF2-S10 phosphorylation under physiological- and post-KA conditions. Thus, we analyzed the correlation between F-actin content and NF2 expression/phosphorylation levels within control siRNA- and NF2 siRNA-treated groups of WT, *PLPP/CIN*^*Tg*^ and *PLPP/CIN*^*−/−*^ mice under physiological conditions, instead of the direct manipulation of PKA activity. Linear regression analysis showed a direct proportional relationship between NF2 expression and F-actin contents with linear correlation coefficients of 0.4237 (*t*_(54)_ = 3.44, *p* = 0.001; Fig. 5a). The F-actin contents also showed a direct proportional relationship with NF2-S10 phosphorylation level (linear correlation coefficients, 0.7977; *t*_(54)_ = 9.72, *p* < 0.001; Fig. 5a). However, the NF2-S518 phosphorylation showed no proportional relationship with F-actin contents (linear correlation coefficients, 0.1975; *t*_(54)_ = 1.48, *p* = 0.1446; Fig. 5a). These findings indicate that S10 phosphorylation may reinforce NF2-mediated F-actin stability independent of cofilin activity.

### NF2 knockdown enhances KA-induced seizure intensity and its progression

**Fig. 6:**
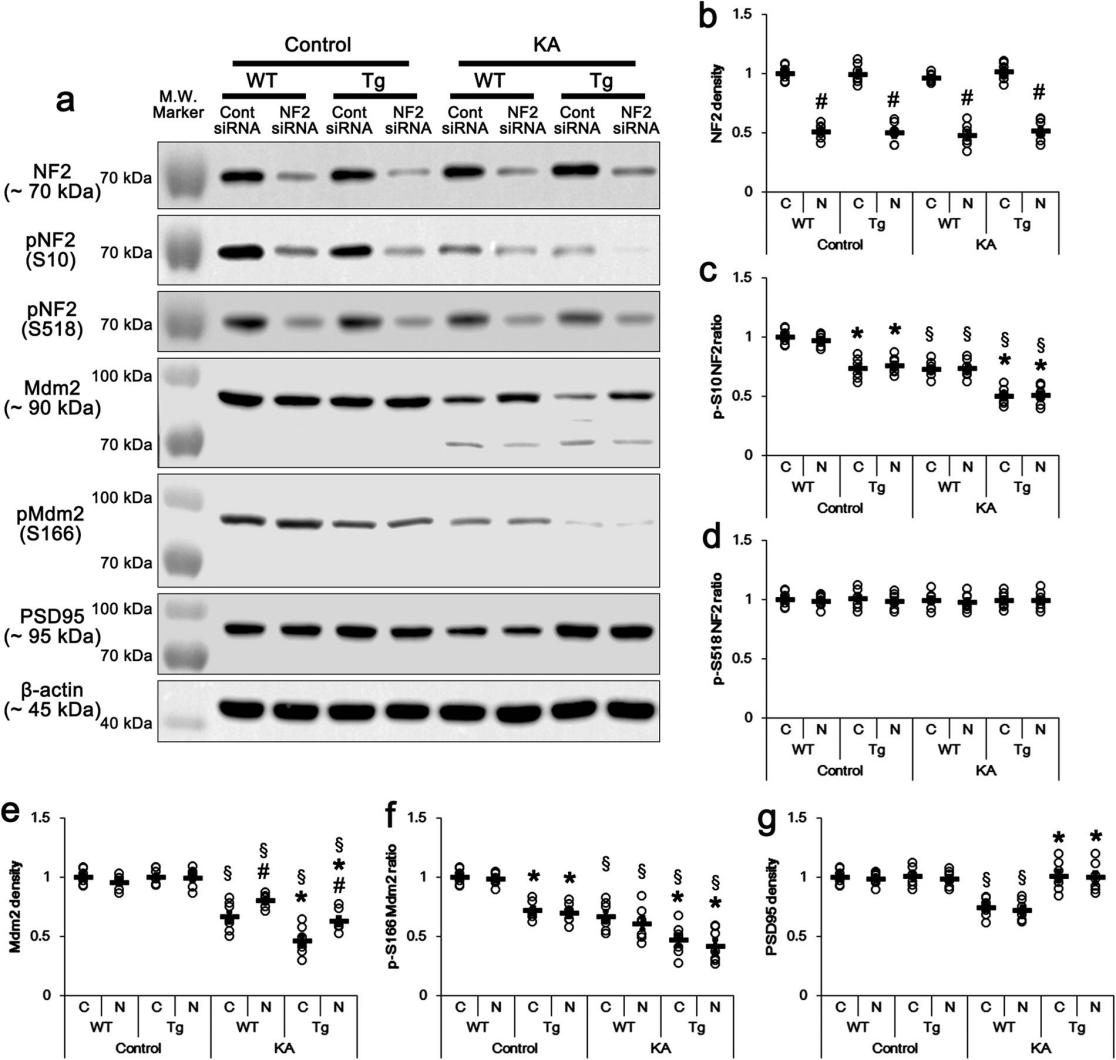
Effects of NF2 knockdown on protein and phosphorylation levels of NF2, Mdm2, and PSD95 in *PLPP/CIN*^*Tg*^ mice following KA injection. **a** Representative western blots of NF2, pNF2-S10, pNF2-S518, Mdm2, pMdm2-S166, and PSD95. **b–g** Quantification of NF2, pNF2-S10, pNF2-S518, Mdm2, pMdm2-S166, and PSD95 levels based on western blot data. Open circles indicate each individual value. Horizontal bars indicate mean value (*^,#,$^*p* < 0.05 vs. WT, control siRNA- and control (non-KA-treated) animals, respectively; *n* = 7).

**Fig. 7:**
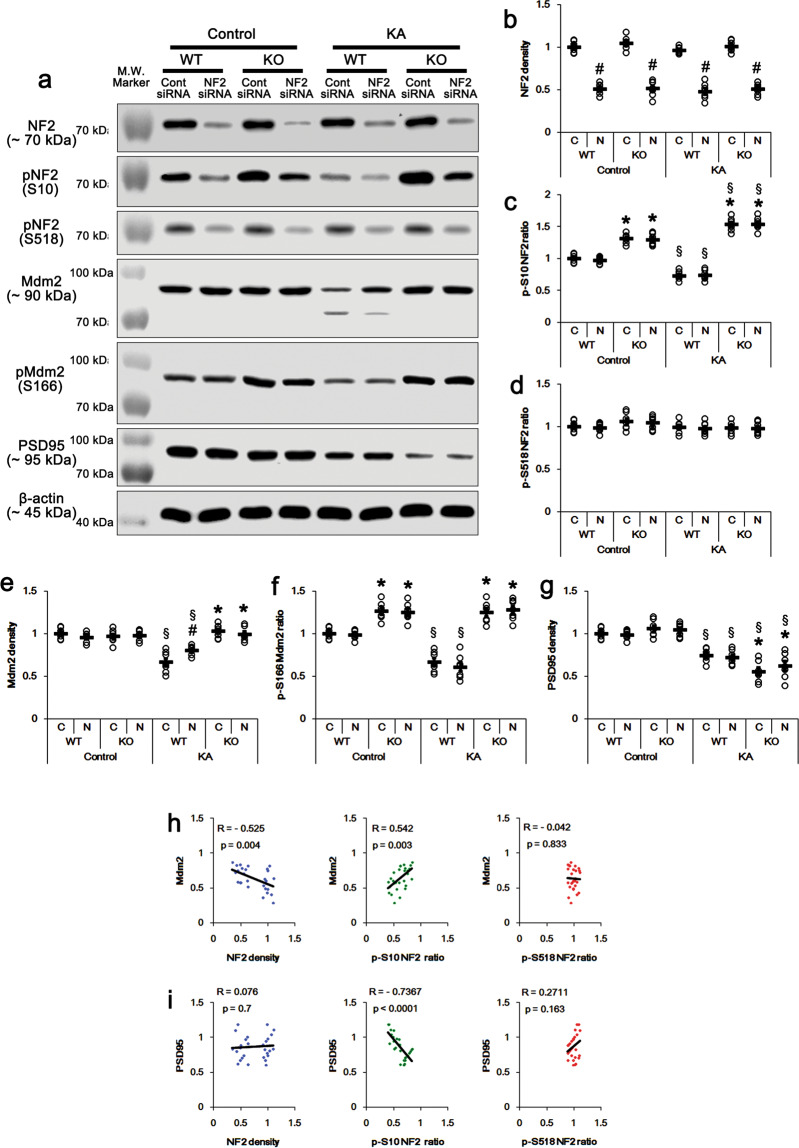
Effects of NF2 knockdown on protein and phosphorylation levels of NF2, Mdm2, and PSD95 in *PLPP/CIN*^*−/−*^ mice following KA injection, and linear regression analysis between NF2 and Mdm2/PSD95 levels. **a** Representative western blots of NF2, pNF2-S10, pNF2-S518, Mdm2, pMdm2-S166, and PSD95. **b–g** Quantification of NF2, pNF2-S10, pNF2-S518, Mdm2, pMdm2-S166, and PSD95 levels based on western blot data. Open circles indicate each individual value. Horizontal bars indicate mean value (*^,#,$^*p* < 0.05 vs. WT, control siRNA- and control (non-KA-treated) animals, respectively; *n* = 7). **h, i** Linear regression analysis between NF2 and Mdm2/PSD95 levels within control siRNA- and NF2 siRNA-treated groups of WT and *PLPP/CIN*^*Tg*^ mice following KA injection.

Aberrant F-actin disruption increases seizure susceptibility^12,41^. Recently, we have reported that PLPP/CIN-mediated F-actin depolymerization directly affects KA-induced seizure intensity without altering the latency of seizure on-set. Briefly, jasplakinolide (an F-actin stabilizer) significantly attenuated seizure intensity (total EEG power) in *PLPP/CIN*^*Tg*^ mice, while latrunculin A (an F-actin depolymerizer) increased it in *PLPP/CIN*^*−/−*^ animals^34^. Therefore, it is likely that NF2 knockdown may increase seizure activity in response to KA, if NF2 itself and/or its S10 phosphorylation level enhance F-actin stability. As compared to control siRNA, NF2 siRNA did not affect the latency of seizure on-set in response to KA (Fig. 5b–g). However, NF2 knockdown increased seizure intensity in response to KA in WT and *PLPP/CIN*^*Tg*^ mice (*F*_(1,12)_ = 7.37 and 11.21, *p* = 0.02 and 0.006, repeated measures one-way ANOVA, *n* = 7, respectively; Fig. 5b–e). NF2 siRNA also enhanced KA-induced seizure intensity and facilitated seizure progression in response to KA in *PLPP/CIN*^*−/−*^ mice (*F*_(1,12)_ = 6.13, *p* = 0.03, repeated measure one-way ANOVA, *n* = 7, respectively; Fig. 5f, g). The efficacies of NF2 knockdown on KA-induced seizure activity were *PLPP/CIN*^*Tg*^ > WT > *PLPP/CIN*^*−/−*^ mice (*F*_(5,36)_ = 2.86, *p* = 0.03, repeated measures two-way ANOVA, *n* = 7, respectively; Fig. 5f, g). As compared to control siRNA, NF2 knockdown did not affect NF2 expression and the ratios of NF2 phosphorylations in WT, *PLPP/CIN*^*Tg*^ and *PLPP/CIN*^*−/−*^ mice following KA injection (Figs. 6a–d and 7a–d). Regarding the effects of NF2 knockdown on cofilin activity and F-actin contents, our findings suggest that NF2 down-regulation and/or PLPP/CIN-mediated NF2-S10 dephosphorylation may facilitate seizure intensity and its progress through F-actin instability independent of cofilin-mediated actin dynamics.

### S10 phosphorylation facilitates NF2-mediated Mdm2 degradation in an activity-dependent manner

In the present study, KA decreased NF-S10 phosphorylation, which was reversed by PLPP/CIN deletion (Fig. 3). The N-terminal region (1-130) of NF2 is responsible for the direct NF2-mediated Mdm2 degradation^39^. Furthermore, Mdm2 degrades PSD95 during activity-dependent synapse elimination^36,37^. Recently, we have reported that PLPP/CIN dephosphorylates Mdm2 at S166 site in activity-dependent manners, which inhibits Mdm2-mediated PSD95 degradation by facilitating Mdm2 ubiquitination^38^. Thus, it is plausible that PLPP/CIN-mediated NF2-S10 dephosphorylation would affect the inhibitory effect of Mdm2 on PSD95 protein level. Under physiological condition, neither PLPP/CIN deletion nor its over-expression affected Mdm2 protein level (Figs. 6a, e and 7a, e). However, Mdm2-S166 phosphorylation levels in *PLPP/CIN*^*Tg*^ and *PLPP/CIN*^*−/−*^ mice were 0.7- and 1.3-fold of WT mice level, respectively (*F*_(3,24)_ = 57.21, *p* < 0.0001, one-way ANOVA, *n* = 7, respectively; Figs. 6a, f, 7a and f). The PSD95 protein level was not influenced by PLPP/CIN deletion or over-expression (Figs. 6a, g and 7a, g). NF2 knockdown did not affect Mdm2 protein/phosphorylation level and PSD95 protein level under physiological condition (Figs. 6a, e, f and 7a, e, f).

KA decreased the Mdm2 protein level to 0.66- and 0.46-fold of control WT levels in WT mice and *PLPP/CIN*^*Tg*^ mice, respectively (*F*_(3,24)_ = 30.98, *p* < 0.0001, one-way ANOVA, *n* = 7, respectively; Figs. 6a, e and 7a, e). KA also reduced Mdm2-S166 phosphorylation to 0.66- and 0.47-fold of control WT levels in WT mice and *PLPP/CIN*^*Tg*^ mice, respectively (*F*_(3,24)_ = 52.87, *p* < 0.0001, one-way ANOVA, *n* = 7, respectively; Fig. 6a and f). NF2 siRNA attenuated KA-induced reduction in the Mdm2 protein level, but not its S166 phosphorylation, in WT and *PLPP/CIN*^*Tg*^ mice (*F*_group(1,24)_ = 24.69, *p* < 0.0001; *F*_siRNA(1,24)_ = 16.24, *p* = 0.0004; *F*_group*siRNA(1,24)_ = 0.13, *p* = 0.72; two-way ANOVA, *n* = 7, respectively; Fig. 6a, e, f). In *PLPP/CIN*^*−/−*^ mice, KA did not influence the Mdm2 protein level and its S166 phosphorylation, which were unaffected by NF2 siRNA (Fig. 7a, e, f). KA also decreased PSD95 protein level to 0.74- and 0.55-fold of control levels in WT and *PLPP/CIN*^*−/−*^ mice, respectively (*F*_(3,24)_ = 23.01, *p* < 0.0001, one-way ANOVA, *n* = 7, respectively; Fig. 7a and g). NF2 knockdown did not affect the KA-induced alterations in PSD95 protein level in WT and *PLPP/CIN*^*−/−*^ mice (Figs. 6a, g and 7a, g). In *PLPP/CIN*^*Tg*^ mice, KA did not affect PSD95 protein level, which was unaltered by NF2 knockdown (Fig. 6a, g).

In the presence of PLPP/CIN (in WT and *PLPP/CIN*^*Tg*^ mice), furthermore, linear regression analysis showed an inverse proportional relationship between NF2 expression and Mdm2 protein level with linear correlation coefficients of −0.525 within control siRNA- and NF2 siRNA-treated groups following KA injection (*t*_(26)_ = 3.145, *p* = 0.004; Fig. 7h). The Mdm2 protein level showed a direct proportional relationship with NF2-S10 phosphorylation after KA treatment (linear correlation coefficients, 0.542; *t*_(26)_ = 3.289, *p* = 0.003; Fig. 7h). However, the NF2-S518 phosphorylation showed no proportional relationship with Mdm2 protein level (linear correlation coefficients, −0.042; *t*_(26)_ = 0.213, *p* = 0.833; Fig. 7h). Furthermore, NF2-S10 phosphorylation, but not NF2 protein and S518 phosphorylation levels, showed an inverse proportional relationship with PSD95 protein level with linear correlation coefficients of −0.7367 within control siRNA- and NF2 siRNA-treated groups under post-KA conditions (*t*_(26)_ = 5.555, *p* < 0.0001; Fig. 7i). These findings indicate that NF2-S10 dephosphorylation by PLPP/CIN may inhibit Mdm2-mediated PSD95 degradation.

### NF2 knockdown deteriorates seizure-induced neuronal death in the CA3 region

**Fig. 8:**
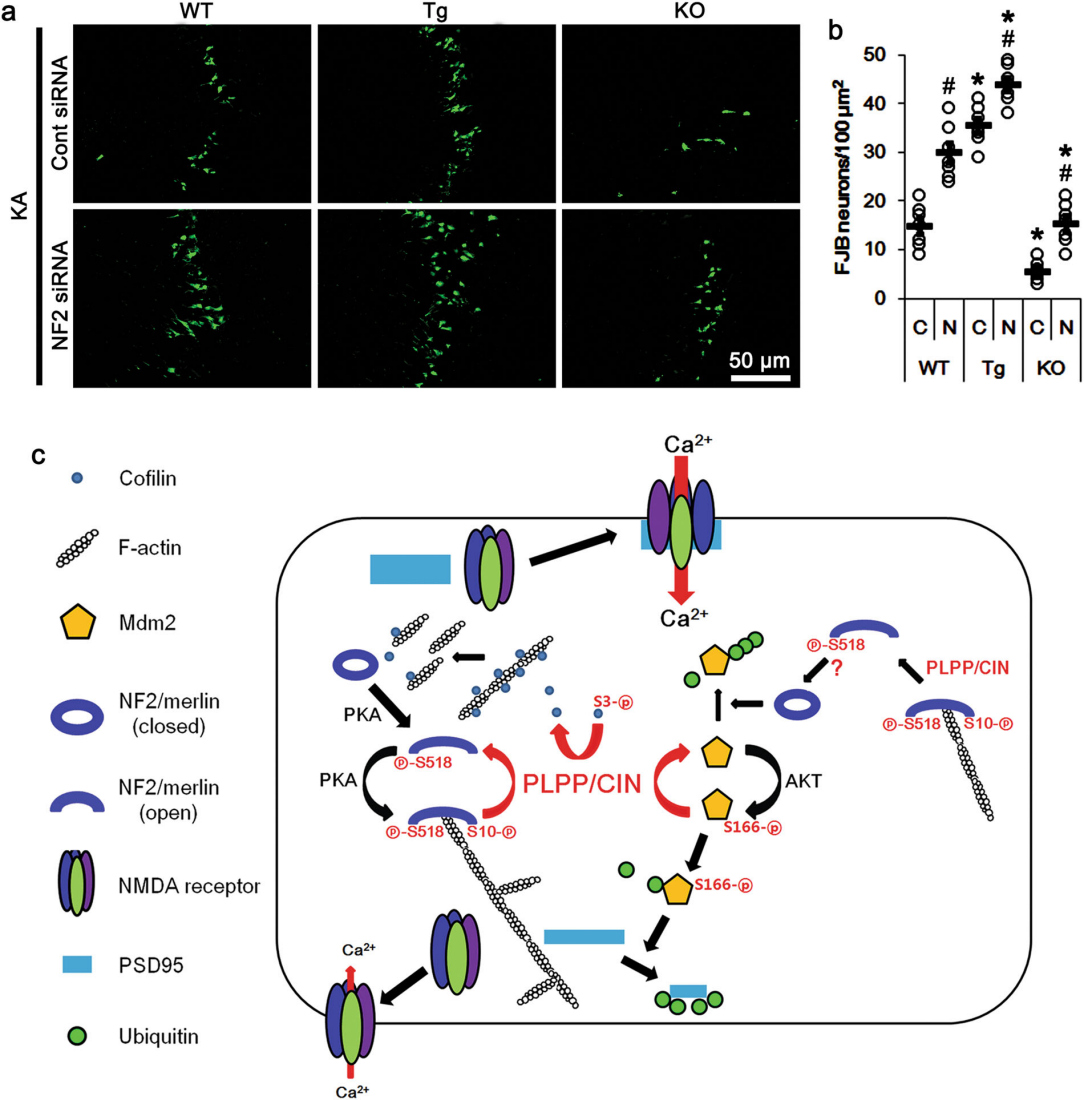
Effects of NF2 knockdown on CA3 neuronal death in WT, *PLPP/CIN*^*Tg*^, and *PLPP/CIN*^*-/*^ mice following KA injection and the role of PLPP/CIN in NF2-S10 and Mdm2-S166 dephosphorylation. **a** Representative photos of FJB-positive degenerating neurons in the CA1 region. **b** Quantification of the number of FJB-positive neurons in response to KA. Open circles indicate each individual value. Horizontal bars indicate mean value (*^,#^*p* < 0.05 vs. WT and control siRNA-treated animals; *n* = 7, respectively). **c** Scheme of the role of PLPP/CIN in NF2-S10 and Mdm2-S166 dephosphorylation. PLPP/CIN-mediated NF2 dephosphorylation decreases F-actin stability and increases NMDAR-PSD95 co-assembly by eliminating the local F-actin barrier. PLPP/CIN also inhibits Mdm2 activity as an E3 ubiquitin ligase for PSD95 by enhancing its S166 dephosphorylation and NF2-mediated Mdm2 degradation.

To confirm the role of NF2 knockdown in seizure activity, we evaluated the effect of NF2 siRNA on KA-induced CA3 neuronal death by Fluoro-Jade B (FJB) staining. Consistent with the seizure activity in response to KA, *PLPP/CIN*^*Tg*^ mice showed profound CA3 pyramidal neuronal damage as compared to WT mice, while *PLPP/CIN*^*−/−*^ mice demonstrated the overt attenuation of neuronal damage in these neurons (*F*_(2,18)_ = 128.84, *p* < 0.0001, one-way ANOVA, *n* = 7, respectively; Fig. 8a, b). These findings indicate that PLPP/CIN may regulate seizure-induced neuronal death by increasing seizure intensity. NF2 siRNA exacerbated seizure-induced CA3 neuronal damage in WT, *PLPP/CIN*^*Tg*^ and *PLPP/CIN*^*−/−*^ mice (*F*_group(2,36)_ = 179.66, *p* < 0.0001; *F*_siRNA(1,36)_ = 76.79, *p* < 0.0001; *F*_group*siRNA(2,36)_ = 2.64, *p* = 0.09; two-way ANOVA, *n* = 7, respectively; Fig. 8a, b). These findings suggest that the reduced NF2 protein level may also facilitate the seizure progression in response to KA, and worsen seizure-induced neuronal death, independent of PLPP/CIN expression level.

## Discussion

PLPP/CIN is a PLP phosphatase and a serine protein phosphatase, which activates cofilin-mediated F-actin depolymerization^30,31^. Recently, we have reported that PLPP/CIN dephosphorylates NEDD4-2, Mdm2, and calsenilin (CSEN, also known as downstream regulatory element antagonist modulator or potassium channel interacting protein 3), independent of cofilin-mediated F-actin depolymerization^34,35,38^. In the present study, we found that PLPP/CIN also dephosphorylated NF2 at S10 site under physiological- and post-KA conditions. Furthermore, the NF2 protein level and NF2-S10 phosphorylation level showed a direct proportional relationship with F-actin contents. NF2 is an actin-binding protein with anti-proliferative activity that is regulated by phosphorylations. A closed clamp conformation of NF2 via intramolecular interactions of its N-terminal domain with an α-helical C-terminal domain acts as an active growth suppressor. The phosphorylation of serine 518 by PKA and/or PAK1 leads to an open conformation and inhibits anti-proliferative and actin-binding activities of NF2^24^. In contrast, the first 18 amino acids of NF2 are involved in actin-binding^42^, and PKA-mediated S10 phosphorylation enhances NF2-actin binding and F-actin stability^29^. Given the role of NF2-S10 phosphorylation in F-actin stability^29^, our findings suggest that PLPP/CIN may reduce F-actin stabilization and filament assembly by dephosphorylating NF2 and cofilin at S10 and S3 site, respectively.

While NF2 selectively binds and stabilizes actin filaments^21,43^, loss of NF2 function paradoxically abrogates F-actin disassembly by increasing LIMK1-mediated cofilin inactivation^22,23^. In the present study, NF2 knockdown reduced F-actin contents in *PLPP/CIN*^*Tg*^ and *PLPP/CIN*^*−/−*^ mice, although it did not affect p-S10 NF2 ratio and cofilin phosphorylation in both groups. In addition, the NF2 protein level and the p-S10 NF2 ratio showed a direct proportional relationship with F-actin contents. Since PLPP/CIN over-expression and its deletion do not change LIMK1 activity^11^, these findings suggest that NF2 itself may interfere with cofilin-actin bindings and/or reduce the yield of cofilin-mediated F-actin depolymerization, independent of LIMK1 activity.

F-actin is one of the most abundant cytoskeletal proteins in dendritic spines, which regulates spine morphogenesis and synaptic strength^44,45^. Indeed, F-actin depolymerization in dendritic spines increases neuronal excitability and decreases seizure thresholds^12,32^. In our previous study^34^, *PLPP/CIN*^*Tg*^ mice show the more severe seizure intensity and the prolonged seizure progression in response to KA, while *PLPP/CIN*^*Tg*^ mice interrupt these phenomena. Furthermore, jasplakinolide (an F-actin stabilizer) ameliorates seizure intensity (total EEG power) in *PLPP/CIN*^*Tg*^ mice, while latrunculin A (an F-actin depolymerizer) aggravates it in *PLPP/CIN*^*−/−*^ animals. Thus, we have reported that PLPP/CIN-mediated F-actin depolymerization contributes to the seizure intensity and its progression in response to KA. Similar to latrunculin A treatment, the present study reveals that NF2 knockdown increased seizure intensity in response to KA in WT, *PLPP/CIN*^*Tg*^, and *PLPP/CIN*^*−/−*^ mice, and its efficacies on seizure activity were *PLPP/CIN*^*Tg*^ > WT > *PLPP/CIN*^*−/−*^ mice. Considering the effects of NF2 knockdown on its phosphorylation ratios, F-actin contents, and cofilin activity, these findings indicate that NF2 itself and/or NF2-S10 phosphorylation may attenuate seizure activity in response to KA through F-actin stability, independent of cofilin activity. Therefore, our findings suggest that PLPP/CIN-mediated NF2 dephosphorylation may serve as one of the mechanisms by which F-actin instability mediates neuronal hyperexcitability.

On the other hand, neuronal hyperexcitability triggers the Mdm2-mediated p53 degradation and the subsequent NEDD4-2 induction as adaptive responses^46,47^, although the roles of p53 in seizure activity have been still controversial^48–52^. The stabilization of p53 by NF2 is accomplished through Mdm2 degradation and the N-terminal region of NF2 is responsible for this activity^39,53^. Therefore, we hypothesized that NF2 would attenuate KA-induced seizure activity by regulating Mdm2-p53 signaling pathway, although little is currently known regarding the contribution of NF2-Mdm2 axis to the neuronal excitability. In the present study, NF2 knockdown attenuated KA-induced Mdm2 degradation in WT and *PLPP/CIN*^*Tg*^ mice, which was abrogated by PLPP/CIN deletion. Aforementioned, however, the present data show that NF2 siRNA exacerbated KA-induced seizure activity, similar to the cases of Mdm2 knockdown^38^. Since both PLPP/CIN and Mdm2 do not affect protein level of p53 in post-mitotic neurons under physiological- and post-KA conditions^38^, it is likely that NF2 may regulate seizure activity independent of the Mdm2–p53 signaling pathway.

Consistent with a previous study^38^, the present study demonstrates that PLPP/CIN dephosphorylated Mdm2-S166 site, and facilitated its degradation induced by KA injection. Furthermore, KA increased NF-S10 dephosphorylation, which was exerted by PLPP/CIN over-expression. Interestingly, NF2 also decreases Mdm2-S166 phosphorylation by inhibiting AKT activity^54^. Thus, it is plausible that NF2 itself and/or NF2-S10 phosphorylation would directly affect AKT-mediated Mdm2-S166 phosphorylation. However, the present data show that NF2 siRNA did not change Mdm2-S166 phosphorylation under physiological condition. Furthermore, PLPP/CIN over-expression and its deletion do not affect AKT activity under physiological- and post-KA conditions^38^. Therefore, it is excluded the possibility that the NF2-mediated AKT regulation would affect Mdm2-S166 phosphorylation in *PLPP/CIN*^*Tg*^ and *PLPP/CIN*^*−/−*^ mice.

KA-induced seizure activity decreases PSD95 levels in the hippocampus in vivo^55,56^, and promotes its translocation from spines to dendritic shafts^57^. Although PSD95 does not govern synaptic currents, subunit expression, and localization of NMDAR, it modulates the gating, trafficking, and intracellular signal pathways of intact NMDAR^58–60^. Since Mdm2 ubiquitinates PSD95 during activity-dependent synapse elimination^36,37^, PSD95 degradation may be an adaptive response to inhibit seizure activity. Indeed, PLPP/CIN dephosphorylates Mdm2 at S166 site in activity-dependent manners, which inhibits PSD95 degradation by facilitating Mdm2 ubiquitination^38^. In the present study, NF2 knockdown did not affect Mdm2-mediated PSD95 degradation induced by KA, although it increased the Mdm2 protein level. Considering no effect of NF2 knockdown on Mdm2-S166 phosphorylation, these findings indicate that Mdm2-S166 phosphorylation may be required for Mdm2-mediated PSD95 degradation. The present study also demonstrates that following KA injection NF2 protein level and NF2-S10 phosphorylation showed an inverse and a direct proportional relationship with the Mdm2 protein level in the presence of PLPP/CIN, respectively. Furthermore, the NF2-S10 phosphorylation level, but not the NF2 protein and S518 phosphorylation levels, showed an inverse proportional relationship with the PSD95 protein level. Regardless of the roles of AKT, p53 and NEDD4-2 in seizure activity, our findings suggest that PLPP/CIN-mediated NF2-S10 dephosphorylation may increase KA-induced seizure activity by enhancing F-actin instability and Mdm2-mediated PSD95 degradation (Fig. 8c).

F-actin acts as an anchor for PSD scaffolding proteins^61,62^. Furthermore, F-actin forms obstacles and barriers for the flux of synaptic molecules, which influence synaptic activity^63^. Indeed, F-actin lattice hinders the redistribution and translocations of postsynaptic proteins, and the receptor-bindings with PSD-related regulatory molecules including PSD95 (refs. ^8,11,61^). With respect to these reports, it is likely that PLPP/CIN may eliminate the local F-actin barrier by dephosphorylations of cofilin and NF2, which may provide a critical window of opportunity allowing translocations and interactions between NMDAR and PSD95. Indeed, *PLPP/CIN*^*Tg*^ mice show the enhanced NMDAR functionality by increasing NMDAR-PSD95 co-assembly^11^. In addition, PLPP/CIN may inhibit Mdm2 activity as an E3 ubiquitin ligase for PSD95 by enhancing its S166 dephosphorylation and NF2-mediated Mdm2 degradation. These PLPP/CIN functions may increase NMDAR-mediated neuronal excitability, which would lead to the enhanced seizure intensity and its progression (Fig. 8c).

As previously stated, PLPP/CIN also regulates seizure progression and intensity in response to KA through Mdm2- and NEDD4-2-medaited PSD95 and AMPAR GluA1 subunit ubiquitination, respectively^35,38^. Indeed, knockdown of NEDD4-2 or Mdm2 increases KA-induced seizure intensity in *PLPP/CIN*^*−/−*^ mice without affecting the latency of seizure on-set^35,38^. Similar to the effect of latrunculin A on seizure intensity^34^, the present study shows that NF2 knockdown enhanced KA-induced seizure intensity, but not latency of seizure on-set, and facilitated seizure progression in response to KA in *PLPP/CIN*^*−/−*^ mice. These findings indicate that PLPP/CIN-mediated dephosphorylations of NEDD4-2, Mdm2, and NF2 may play important roles in seizure progression and propagation in response to KA. However, PLPP/CIN-mediated CSEN dephosphorylation reduces seizure susceptibility by activating Kv4.2 channel (an A-type K^+^ channel), and increases seizure duration and its intensity via prolonged NMDAR activation following KA^34^. These diverse roles of PLPP/CIN in KA-induced seizure activity suggest that the seizure susceptibility (initiation) and its severity (progression) may be regulated by different and complicated mechanisms. Together with these previous studies, the present data also hypothesize that PLPP/CIN may be one of the up-stream regulators for the various signaling molecules participating the ictogenesis and seizure progression.

In conclusion, the present data provide a new implication for the interaction between the PLPP/CIN and NF2: PLPP/CIN-mediated NF2-S10 dephosphorylation may serve as one of the mechanisms by which F-actin instability induces neuronal hyperexcitability, and Mdm2 may act as the mediator of the interaction between NF2 and PSD95. To our knowledge, this is the first report concerning the possible PLPP/CIN–NF2–Mdm2–PSD95 signaling pathway, which may lead to seizure progression and increase its severity, independent of cofilin activity and p53–NEDD4-2 axis. Therefore, we provide a new paradigm for the development of therapeutic strategies for epilepsy and neurological diseases associated with deregulation of NF2 and Mdm2.

## Materials and methods

### Experimental animals and chemicals

Male *PLPP/CIN*^*−/−*^ (129/SvEv-C57BL/6J background) and *PLPP/CIN*^*Tg*^ (C57BL/6J background) mice (8 weeks old) were used in the present study. Each background WT mice were used as control animals for *PLPP/CIN*^*−/−*^ and *PLPP/CIN*^*Tg*^ mice, respectively. Animals were provided with a commercial diet and water ad libitum under controlled temperature, humidity, and lighting conditions (22 ± 2 °C, 55 ± 5% and a 12:12 light/dark cycle). All experimental protocols were approved by the Animal Care and Use Committee of Hallym University (# Hallym 2018-2, 26 April 2018). All reagents were obtained from Sigma-Aldrich (USA), except as noted.

### In vitro PLPP/CIN phosphatase assay

Modified in vitro PLPP/CIN phosphatase assay using full-length recombinant human NF2 (#MBS956203, MyBiosource.com., USA) and human PLPP/CIN proteins (ab97953, Abcam, UK) was performed as described previously^34,35,38^. NF2 (10 ng/μl) was phosphorylated by incubation with 200 U/μl active PKAc (GST tag, #P51-10G, SignalChem, Canada) and 100 μM ATP in kinase assay buffer I (#K01-09, SignalChem, Canada) at 30 °C for 1 h. Thereafter, the sample was portioned the same volume, added PLPP/CIN (10 ng/μl) or 50 mM Tris buffer (control) and incubated at 30 °C for 1 h. The mixture was used for co-immunoprecipitation and western blot analysis (see below).

### Analysis of F-actin content

To analyze F-actin content, we used G-actin/F-actin in vivo assay biochem kit (#BK037, Cytoskeleton, Inc., USA), according to the manufacturer’s instructions^11^. Next, western blotting was performed according to the standard procedures (see below).

### NF2 knockdown and electrode implantation

Under anesthesia with isoflurane (3% induction, 1.5–2% for surgery, and 1.5% maintenance in a 65:35 mixture of N_2_O:O_2_). Surgery for a brain infusion kit and an electrode implantation was performed according to our previous study^34,35,38^. A brain kit 3 (Alzet, USA) was inserted into the lateral cerebral ventricle (2.0 mm depth from bregma), and connected with a 1007D Alzet osmotic pump (Alzet, USA) containing control siRNA (20 μM) or NF2 siRNA (20 μM), respectively. siRNA sequence targeting NF2 corresponding to coding region (5′-CUGAUCAGUUAAAGCAAGAtt-3′ or 5′-UCUUGCUUUAACUGAUCAGtt-3′). Non-silencing RNA (5′-GGCGCGCTTTGTAGGATTCGA-3′) was used as the control siRNA (Bioneer, South Korea). Osmotic pump was implanted subcutaneously in the midscapular region of the back. Monopolar electrode (Plastics One, USA) or a guide-electrode-combo (C313G-MS303/2/SPC, Plastics One, USA) was also implanted into the left dorsal hippocampus (2 mm posterior; 1.25 mm lateral; 2 mm depth from bregma).

### Seizure induction and EEG recording

After baseline recording for at least 30 min, animals were given KA (25 mg/kg, i.p.). Control animals received an equal volume of normal saline instead of KA. EEG signals were recorded with a DAM 80 differential amplifier (0.1–1000 Hz bandpass; World Precision Instruments, USA) and the data were digitized (1000 Hz) and analyzed using LabChart Pro v7 software (AD Instruments, Australia). Latency of seizure on-set was defined as the time point showing more than 3 s and consisting of a rhythmic discharge between 4 and 10 Hz with an amplitude of at least two times higher than the baseline EEG^34,35,38^. Total EEG power was normalized by the baseline power obtained from each animal. Spectrograms were automatically calculated using a Hanning sliding window with 50% overlap by LabChart Pro v7. Diazepam (Valium; Roche, France; 10 mg/kg, i.p.) was administered 2 h after KA injection. This is because 2-h after KA injection is the suitable time point to compare the time of seizure on-set, total EEG power and the changes in biochemical profiles in *PLPP/CIN*^*Tg*^ and *PLPP/CIN*^*−/−*^ mice^34,35,38^. Behavioral seizure severity was also evaluated based on the seizure score as followed: (0) no change, (1) no movement, (2) increase in muscle tone at rest, (3) head bobbing/scratching or and circling, (4) clonus/rearing/falling of forelimb, (5) repetitive behavior of 4, (6) severe tonic–clonic seizures^48^. After recording, animals were quickly decapitated, and their hippocampi were dissected out in the presence of cooled artificial cerebrospinal fluid (in mM: 124 NaCl, 5 KCl, 1.25 NaH_2_PO_4_, 26 NaHCO_3_, 10 dextrose, 1.5 MgCl_2_, and 2.5 CaCl_2_) and stored −80 °C until preparation for biochemical experiments^34,35,38^.

### Co-immunoprecipitation

The hippocampal tissues were lysed in radioimmunoprecipitation assay buffer (RIPA: 50 mM Tris–HCl pH 8.0; 1% Nonidet P-40; 0.5% deoxycholate; 0.1% sodium dodecyl sulfate (SDS), Thermo Fisher Scientific, USA) containing protease inhibitor cocktail (Roche Applied Sciences, USA), phosphatase inhibitor cocktail (PhosSTOP^®^, Roche Applied Science, USA), and 1 mM sodium orthovanadate. Protein concentrations were calibrated by BCA protein assay (Pierce, USA) and equal amounts of total proteins (150 μg) were incubated with NF2 or PLPP/CIN antibody (Supplementary Table 1) and protein G sepharose beads at 4 °C overnight. In vitro sample were also reacted with each antibody by the same method. Beads were collected by centrifugation, eluted in 2× SDS sample buffer, and boiled at 95 °C for 5 min. Thereafter, the samples were used for western blot.

### Western blot

Western blotting was performed according to standard procedures. Briefly, sample proteins (10 μg) were separated on a Bis-Tris SDS-polyacrylamide electrophoresis gel (SDS-PAGE). Separated proteins then were transferred to polyvinylidene fluoride membranes. The membranes were incubated with a relatively specific primary antibody (Supplementary Table 1). The ECL Kit (GE Healthcare Korea, Seoul, South Korea) was used to detect signals. The bands were detected and quantified on ImageQuant LAS4000 system (GE Healthcare Korea, Seoul, South Korea). The rabbit anti-β-actin was used as a loading control for quantitative analysis of relative expression levels of proteins. The ratio of phosphoprotein to total protein was described as the phosphorylation ratio.

### FJB staining and cell counting

One day after KA injection, animals were perfused transcardially with 4% paraformaldehyde in 0.1 M phosphate buffer (pH 7.4) under urethane anesthesia (1.5 g/kg, i.p.). Brains were post-fixed in the same fixative overnight and then cryoprotected and sectioned at 30 μm with a cryostat. Thereafter, tissues were used for a conventional Fluoro-Jade B (FJB) staining according to previous studies^34,35,38^. All images were obtained using an AxioImage M2 microscope and AxioVision Rel. 4.8 software. Areas of interest (1 × 10^5^ μm^2^) were selected in the captured images of the CA3 region of the hippocampus proper (10 sections per each animal). Two different investigators who were blind to the classification of tissues performed the cell count of FJB-positive neurons^34,35,38^.

### Statistical analysis

Number (*n*) of each experimental group used for the evaluation was seven. The data obtained from each group were analyzed. After evaluating the values on normality using Shapiro–Wilk *W* test, two-tailed Student’s *t*-test, repeated measures ANOVA, one-way ANOVA, and two-way ANOVA were used to analyze statistical significance. Bonferroni’s test was applied for post hoc comparisons. A *p* value below 0.05 was considered statistically significant.

## Supplementary information

Supplementary Figure 1

Supplementary Figure 2

Supplementary Figure 3

Supplementary Figure 4

Supplementary Figure 5

Supplementary Figure 6

Supplementary Figure Legends
