# Supplementary figures and images for: PLPP/CIN-mediated NF2-serine 10 dephosphorylation regulates F-actin stability and Mdm2 degradation in an activity-dependent manner
Source: Cell Death Dis. 2021 Jan 4;12(1):37. doi: 10.1038/s41419-020-03325-9 (PMC7791067; doi:10.1038/s41419-020-03325-9)

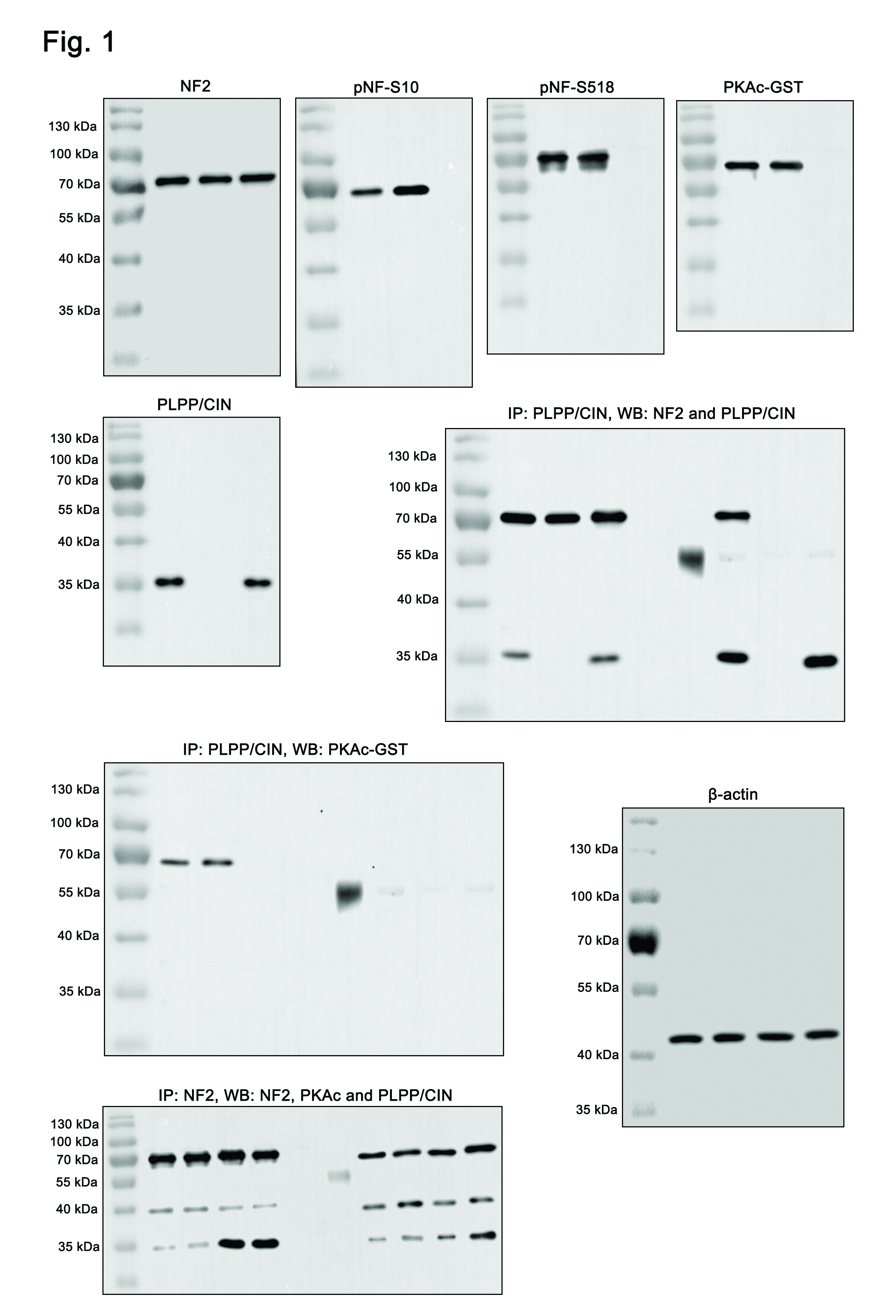

Supplement: Supplementary file 1 — Supplementary Figure 1 [file 41419_2020_3325_MOESM1_ESM.tif]

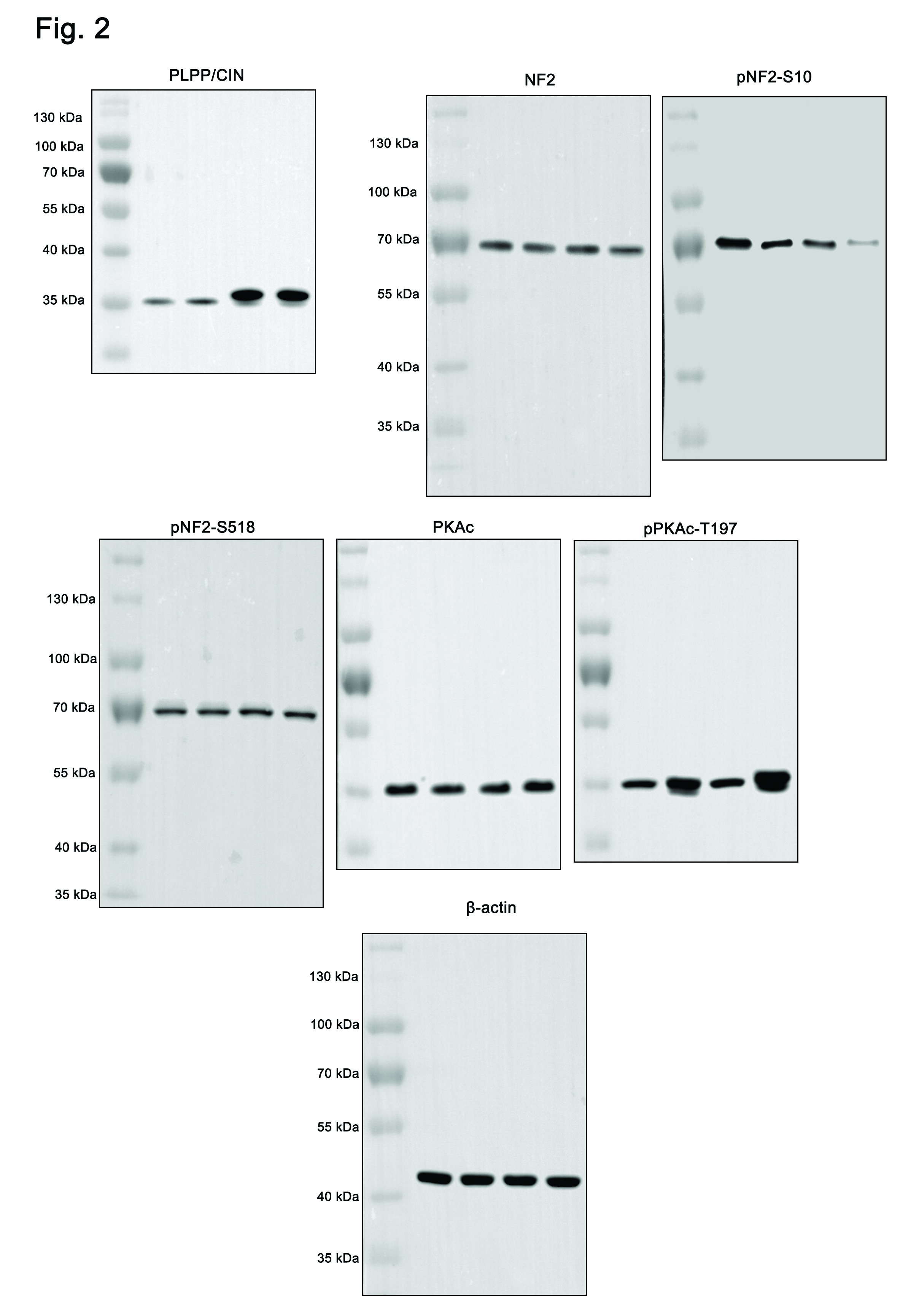

Supplement: Supplementary file 2 — Supplementary Figure 2 [file 41419_2020_3325_MOESM2_ESM.tif]

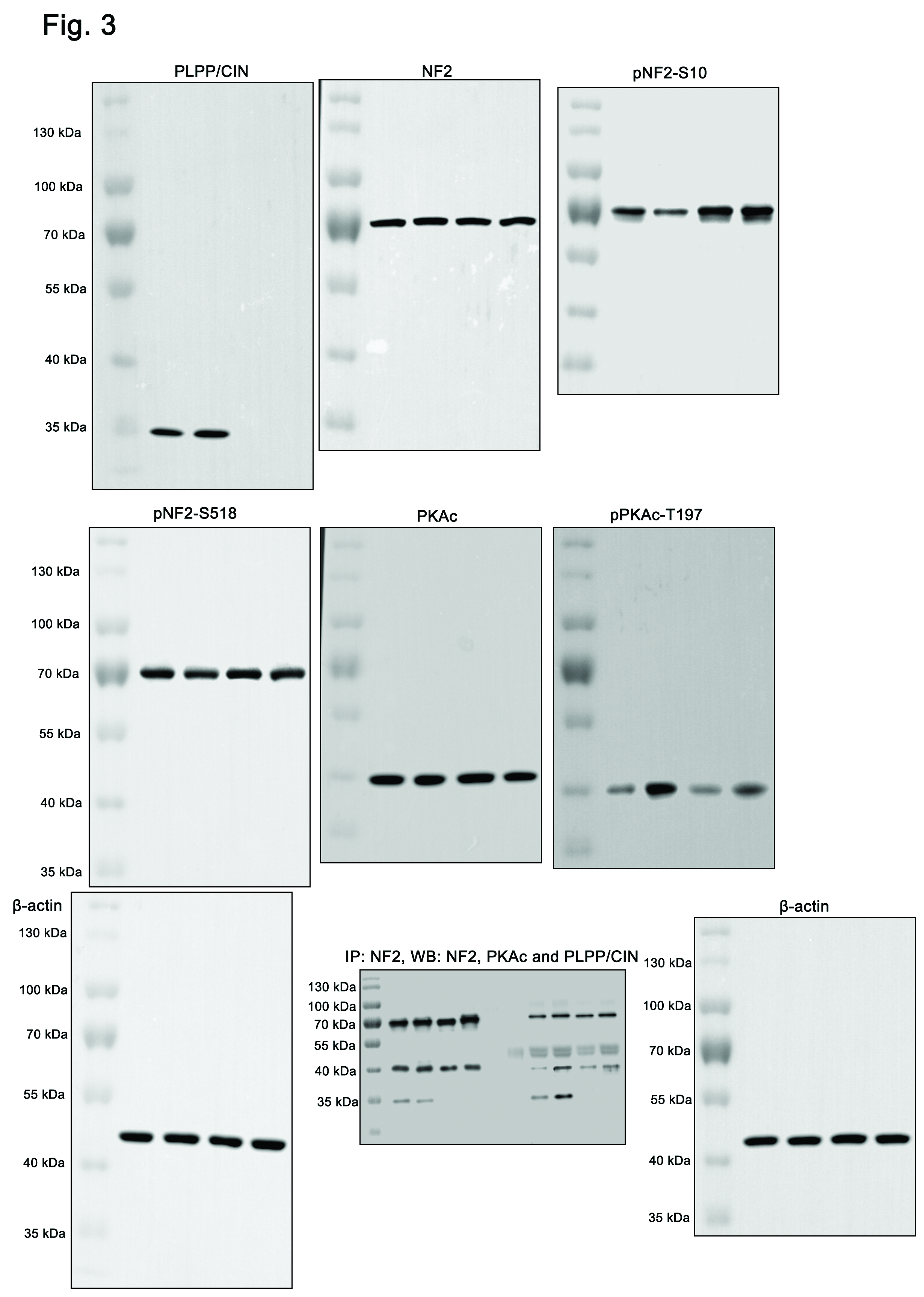

Supplement: Supplementary file 3 — Supplementary Figure 3 [file 41419_2020_3325_MOESM3_ESM.tif]

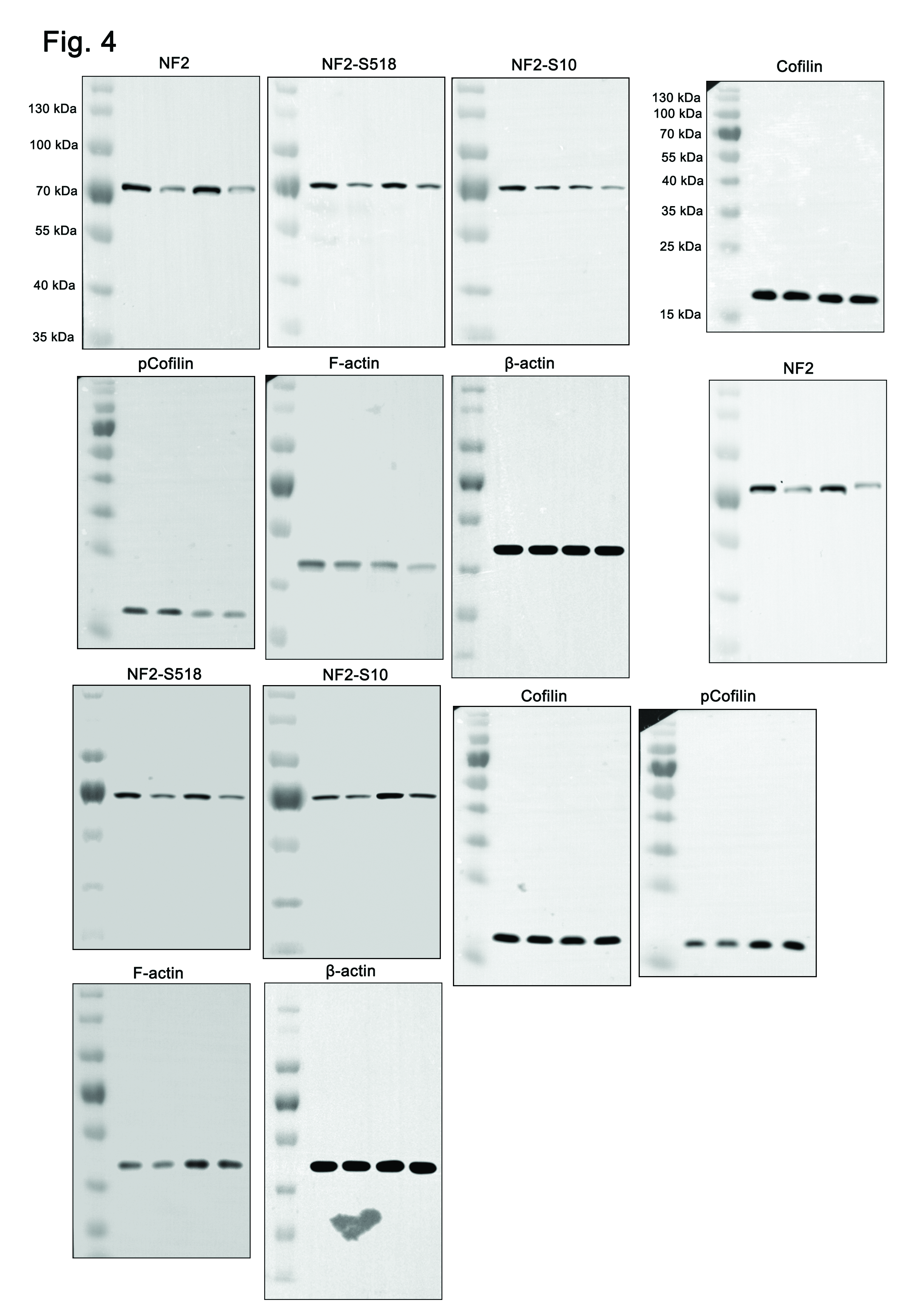

Supplement: Supplementary file 4 — Supplementary Figure 4 [file 41419_2020_3325_MOESM4_ESM.tif]

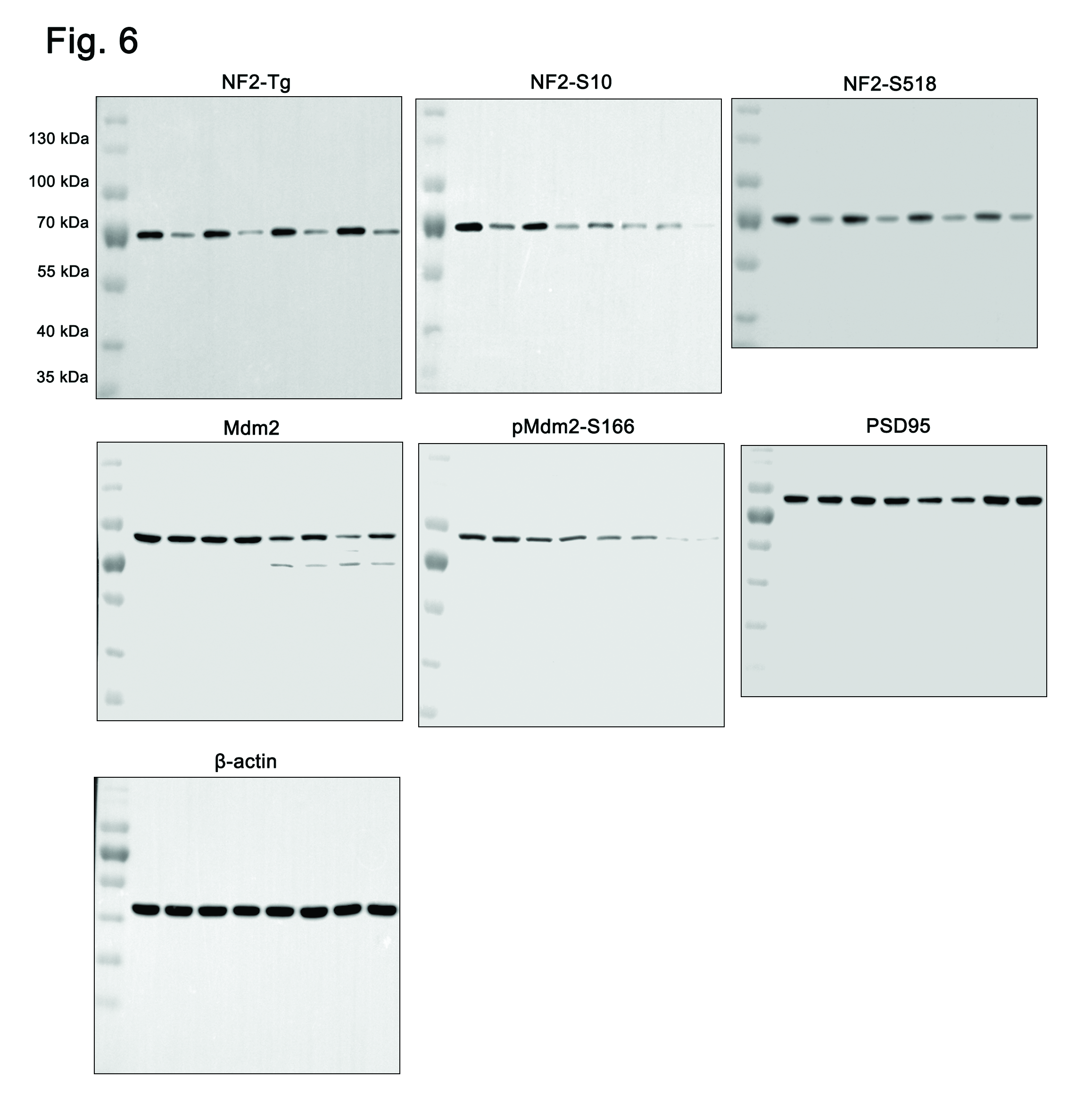

Supplement: Supplementary file 5 — Supplementary Figure 5 [file 41419_2020_3325_MOESM5_ESM.tif]

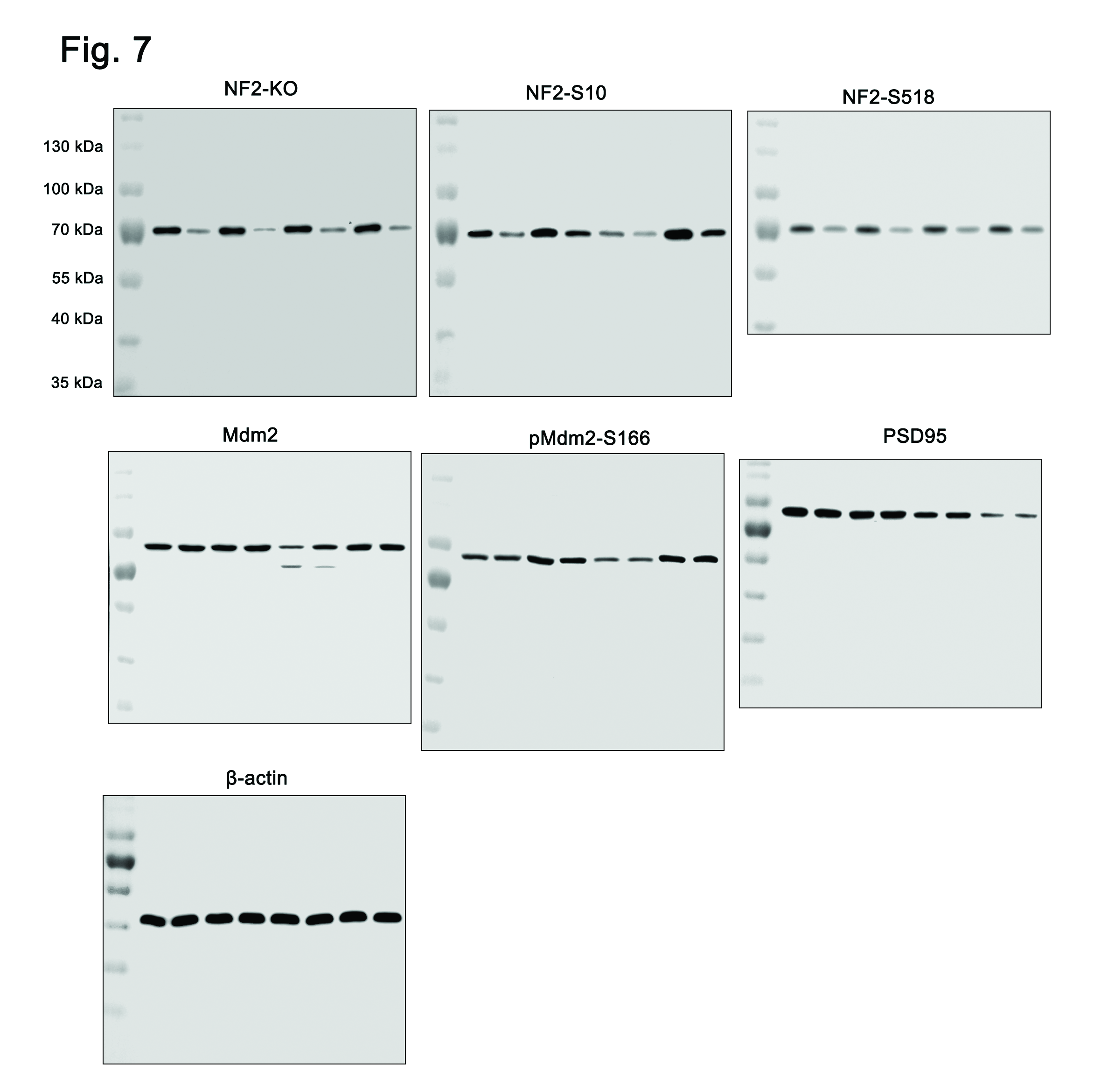

Supplement: Supplementary file 6 — Supplementary Figure 6 [file 41419_2020_3325_MOESM6_ESM.tif]
