# Supplementary material for: PLPP/CIN-mediated NF2-serine 10 dephosphorylation regulates F-actin stability and Mdm2 degradation in an activity-dependent manner
Source: Cell Death Dis. 2021 Jan 4;12(1):37. doi: 10.1038/s41419-020-03325-9 (PMC7791067; doi:10.1038/s41419-020-03325-9)
Supplement: Supplementary file 7 — Supplementary Figure Legends [file 41419_2020_3325_MOESM7_ESM.docx]

**Supplementary Figure Legends**

**PLPP/CIN-mediated NF2-serine 10 dephosphorylation regulates F-actin stability and Mdm2 degradation in an activity-dependent manner**

Ji-Eun Kim,^1^ Duk-Shin Lee,^1^ Tae-Hyun Kim,^1^ Hana Park,^1^ Min-Ju Kim,^1^ Tae-Cheon Kang^1,*^

^1^Department of Anatomy and Neurobiology, Institute of Epilepsy Research, College of Medicine, Hallym University, Chuncheon 24252, South Korea

* Correspondence to: T. -C. Kang, Department of Anatomy and Neurobiology, College of Medicine, Hallym University, Chuncheon, Kangwon-Do 24252, South Korea; Tel: +82-33-248-2524; Fax: +82-33-248-2525; E-mail: tckang@hallym.ac.kr

Running Title: PLPP/CIN-mediated NF2-S10 dephosphorylation

**Supplementary Table 1. Primary antibodies used in the present study**

| Antigen | Host | Manufacturer  (catalog number) | Dilution used |
| --- | --- | --- | --- |
| Cofilin | Rabbit | Sigma (C8736) | 1:10,000(WB) |
| Mdm2 | Mouse | abcam (ab16895) | 1:1000 (WB) |
| NF2 | Rabbit | Elabsacience (ENT3080) | 1:1000 (WB) |
| pCofilin-S3 | Rabbit | Abcam (ab47281) | 1:1000 (WB) |
| PKAc | Rabbit | BioVision (3115-100) | 1:1000 (WB) |
| PLPP/CIN | Rabbit | Santa Cruz (sc-398850)  Sigma (HPA001099) | 1:100 (IP)  1:1000 (WB) |
| pMdm2-S166 | Rabbit | Cell signaling (35215) | 1:1000 (WB) |
| pNF2-S10 | Rabbit | Signalway antibody (#12334) | 1:1000 (WB) |
| pNF2-S518 | Rabbit | Lifespan BioSciences  (LS-C354043) | 1:1000 (WB) |
| pPKAc-T197 | Rabbit | Assay Biotec (A0548) | 1:1,000 (WB) |
| PSD95 | Rabbit | Abcam (ab18258) | 1:1000 (WB) |
| β-actin | Mouse | Sigma (A5316) | 1:5000 (WB) |

IP, Immunoprecipitation; WB, Western blot.

**Supplementary Figure Legends**

Supplementary Fig. 1. Full-length gel images of western blot data in Fig. 1.

Supplementary Fig. 2. Full-length gel images of western blot data in Fig. 2.

Supplementary Fig. 3. Full-length gel images of western blot data in Fig. 3.

Supplementary Fig. 4. Full-length gel images of western blot data in Fig. 4.

Supplementary Fig. 5. Full-length gel images of western blot data in Fig. 6.

Supplementary Fig. 6. Full-length gel images of western blot data in Fig. 7.
